# Training in the Categorization of Aerial and Terrestrial Scenes Differentially Impacts Scene‐Selective and Nonscene‐Selective Regions in Occipitotemporal Cortex

**DOI:** 10.1111/ejn.70599

**Published:** 2026-07-02

**Authors:** Joseph Borders, Birken Noesen, Bethany Dennis, Assaf Harel

**Affiliations:** ^1^ Department of Psychology Wright State University Dayton Ohio USA

**Keywords:** behavior, cognitive neuroscience, perceptual learning, training

## Abstract

Humans are extremely adept at categorizing complex visual environments, an ability supported by a network of scene‐selective cortical areas in occipitotemporal cortex (OTC), primarily parahippocampal‐ and occipital‐place area (PPA, OPA, respectively). Despite increasing knowledge on the development of the scene‐selective network, it is still not well‐understood how experience impacts scene‐related activity in the adult brain. A key question is how activity in scene‐selective cortex changes as people gain experience in categorizing scenes. To address this question, we conducted an fMRI training study focused on the categorization of aerial and terrestrial scenes. Unlike terrestrial scenes, aerial scenes lack the same environmental regularities the brain has adapted to, and thus ideal for testing the impact of experience on scene‐selective cortex. Over six training sessions, 39 participants (19 males and 20 females) were shown scenes of different categories from aerial and terrestrial viewpoints, with half the participants categorizing the scenes at a specific level (e.g., truss bridge/suspension bridge), whereas the other performed an unrelated task on the same images. Both groups were scanned before, during, and after training. We found that categorization training had a group‐specific effect on responses in OPA and PPA, with greater neural sensitivity to viewpoint in the trained versus the untrained group. In contrast, nonscene‐selective regions, such as object‐selective LOC and early visual cortex showed no training effects. Improvements in behavioral performance, including learning transfer, were linked to changes in PPA activity level pre‐ versus‐posttraining. We conclude that scene‐selective cortex can support the learning of novel spatial geometries.

## Graphical abstract

The nature of neuroplasticity in the adult scene‐selective network is poorly understood. Training in categorization of unfamiliar (aerial) scenes results in specific changes to scene‐selective cortex and is predictive of behavioral improvements. We suggest that scene‐selective cortex is malleable and can support the learning of novel spatial geometries.

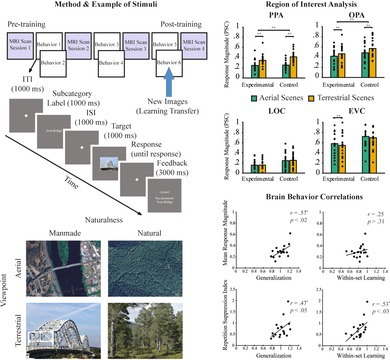

**Abbreviations**
3D: three‐dimensional
AFB: air force base
ANOVA: analysis of variance
BF: Bayes factor
BOLD: blood‐oxygen‐level‐dependent imaging
EPI: echo‐planar imaging
EVC: early visual cortex
fMRI: functional magnetic resonance imaging
FOV: field of view
FWHM: full width at half maximum
GE: general electric
HIPAA: Health Insurance Portability and Accountability Act
IES: inverse efficiency score
IRB: institutional review board
ISI: interstimulus interval
LCD: liquid crystal display
LO: lateral occipital cortex
LOC: lateral occipital complex
M: mean, mm millimeter
ms: millisecond
OPA: occipital place area
OTC: occipitotemporal cortex
PFS: posterior fusiform sulcus
PHC: parahippocampal cortex
PPA: parahippocampal place area
PSC: percent signal change
ROI: region of interest
RS: repetition suppression
RSC: retrosplenial complex
RT: reaction time
SD: standard deviation
SEM: standard error of the mean
TE: echo time
TOS: transverse occipital sulcus
TR: repetition time

## 1 Introduction

Humans are extremely adept at recognizing complex visual scenes (Malcolm et al. 2016), an ability supported by a network of dedicated scene‐selective areas in the occipitotemporal cortex (OTC), such as the parahippocampal place area (PPA) and the occipital place area (OPA) (Epstein and Baker 2019). In spite of a growing understanding of the scene‐selective network, much less is known about how it evolved to deal with the huge computational complexity involved in representing large‐scale spatial environments. Several accounts suggest that throughout evolution the human visual system has adapted to efficiently encode a number of environmental constants (Greene and Oliva 2009; Ringer and Loschky 2018). Since humans are terrestrial animals, these environmental constants reflect a specific ground‐based viewpoint. For example, one adaptive trait of human vision is the strong affinity for detecting horizontal and vertical edges, nested within the perceptual upright layout relative to the horizon (Loschky et al. 2015). Examples include mountains, forests, or city skylines. However, it is still unknown to what extent the scene‐selective cortex can accommodate for new environmental affordances, that is, how flexible is the scene‐selective cortex.^1^ Particularly, what happens when humans leave the terrestrial perspective, for example, when taking a flight, or looking down from a tall building? Are the same neural mechanisms originally evolved for terrestrial scene recognition also recruited for recognizing novel, complex visual environments?

In the present work,^2^ we examine how experience impacts the neural response to complex scenes by training people to recognize aerial scenes and observing the corresponding neural changes. Aerial scenes are scenes that most people have no prior experience with, and as such enable us to explore how the existing neural circuitry supporting everyday (i.e., terrestrial) scene recognition accommodates distinct type of novel scenes. Currently, there is very little research on how people recognize aerial scenes (Lloyd et al. 2002), and even less so on how expertise in aerial scene recognition is developed (Šikl et al. 2019). Prior work suggests that aerial scene recognition is supported by the same areas engaged in terrestrial scene recognition (Shelton and Gabrieli 2002; Barra et al. 2012) yet no study to date has explored how increased experience in aerial scene recognition modulates existing scene‐selective neural circuitry.

To elucidate the neural changes underlying aerial scene recognition and how they compare to terrestrial scene recognition, we asked the following questions: First, how do cortical scene representations change overall with repeated exposure and experience? Second, what are the specific neural signatures of aerial‐ compared to terrestrial scenes? In other words, how does the brain accommodate scenes presented from a novel viewpoint? Third, how does semantic knowledge accumulated through training modulate the underlying perceptual representations in scene‐selective cortex? To address these questions, we conducted a long‐term fMRI study in which we trained naïve participants to categorize scenes presented both in their aerial and terrestrial perspectives and measured their neural activity in response to these same images before, during, and after training. We used manmade and natural scenes to examine whether aerial scene categorization varies as a function of naturalness, a prevalent scene property (Zhang et al. 2019). We focused on how responses to aerial versus terrestrial scenes change over the course of training, to what extent these changes result from general exposure or from the specific training regimen, and lastly, how these changes manifest in scene‐selective cortex (specifically, PPA and OPA) relative to nonscene‐selective areas. Specifically, these nonscene‐selective areas were either object‐selective (lateral occipital complex, LOC: Malach et al. 1995) or general, nonselective early visual cortex (EVC: Roth and Zohary 2015). Extending our previous work on visual expertise (Harel et al. 2013b; Harel 2016), we hypothesized that scene‐selective cortex would be recruited through training to extract information across both aerial and terrestrial scenes. This sensitivity to viewpoint information should be specific to scene‐selective cortex such that changes in its neural responses (but not of other regions) would be directly related to improvements in learning following training.

## 2 Materials and Methods

### 2.1 Subjects

Forty‐six subjects (15 females, age range: 19–34) participated in the study for monetary compensation. The subjects were recruited from the Wright State University community, had normal or corrected‐to‐normal visual acuity, and has no history of psychiatric or neurological disorders. Subjects provided their written informed consent, as well as signed a HIPAA authorization form. The consent form, HIPAA authorization form, and experimental protocol were approved by the Wright State University Institutional Review Board (IRB). All subjects agreed when consenting to participate in six behavioral training sessions and four fMRI scan sessions. Based on a simulation‐based power analysis targeting a medium Group × ROI × Session interaction in a mixed‐design analysis of variance (ANOVA), we set our target sample size in advance to recruit 24 participants per group (48 total). This analysis assumed moderate correlations across repeated measures (ε ≈ 1), an alpha of 0.05, and 80% power. Seven subjects were removed from further analyses due to insufficient behavioral performance (< 80% accuracy) or corrupted behavioral/scanning data (additional subjects who failed to complete the full training and scan regimen were not included in the analysis). Consequently, 39 subjects were used for the analyses reported here. Subjects were randomly assigned to either the control (*n* = 21, 11 females, mean age = 25.57, SD = 3.23) or the experimental group (*n* = 18, 9 females, mean age = 24.45, SD = 3.9). Although short of our target, this sample still offered reasonable power given the repeated‐measures design and multiple within‐subject conditions.

### 2.2 Stimuli

We used a large set of high‐resolution color scene images, consisting of 480 individual images spanning five dimensions: Viewpoint (terrestrial/aerial), Naturalness (manmade/natural), Category (e.g., “airport”), Subcategory (e.g., “military airbase”), and Exemplar (a specific place, e.g., “Edwards AFB”). Each exemplar presented a single place, both in a terrestrial and an aerial viewpoint. We used two viewpoints of the same place to determine the extent to which training in scene categorization involves an increase in the observer's ability to extract invariant information from one viewpoint and apply it to another. The terrestrial viewpoint refers to scene images viewed from the ground and are typical of how someone would view a scene in everyday life. They are often characterized by a horizon and converging perspective lines. The aerial viewpoint refers to a “bird's‐eye view” of a scene. Because of the overhead viewpoint, perspective lines are not present within the aerial images. Terrestrial images were collected from Google Maps, Google Images, Bing Maps, and Bing Images under the Creative Commons License. Aerial images were collected using Bing maps and controlled for altitude (approximately 400–600 ft).

Each subset of aerial and terrestrial images consisted of 240 images: 120 man‐made and 120 natural scenes. Man‐made scenes were defined as scenes with a majority of the content being manufactured or artificial (e.g., buildings, roads, and furniture). Natural scenes were defined as scene images containing mostly natural, organic content (e.g., water, trees, and rocks). We chose to use both man‐made and natural scenes in the study, as naturalness is a fundamental scene property, processed during the early stages of processing (Loschky and Larson 2010; Harel et al. 2016; Lowe et al. 2018) and determines the type of information extracted from the scene (Greene and Oliva 2009; Groen et al. 2013; Harel et al. 2020). We thus examined whether naturalness also plays a role in the extraction of viewpoint information. Specifically, we investigated the extent to which viewpoint information is extracted differentially for natural and man‐made scenes (for a broader discussion of what constitutes natural and man‐made imagery, see Zhang et al. 2019). Note that we chose the term “man‐made” rather than “artificial,” to avoid confusion, as the latter can denote both the opposite of natural (i.e., coming from nature) and the opposite of naturalistic (i.e., not‐synthetic).

**FIGURE 1:**
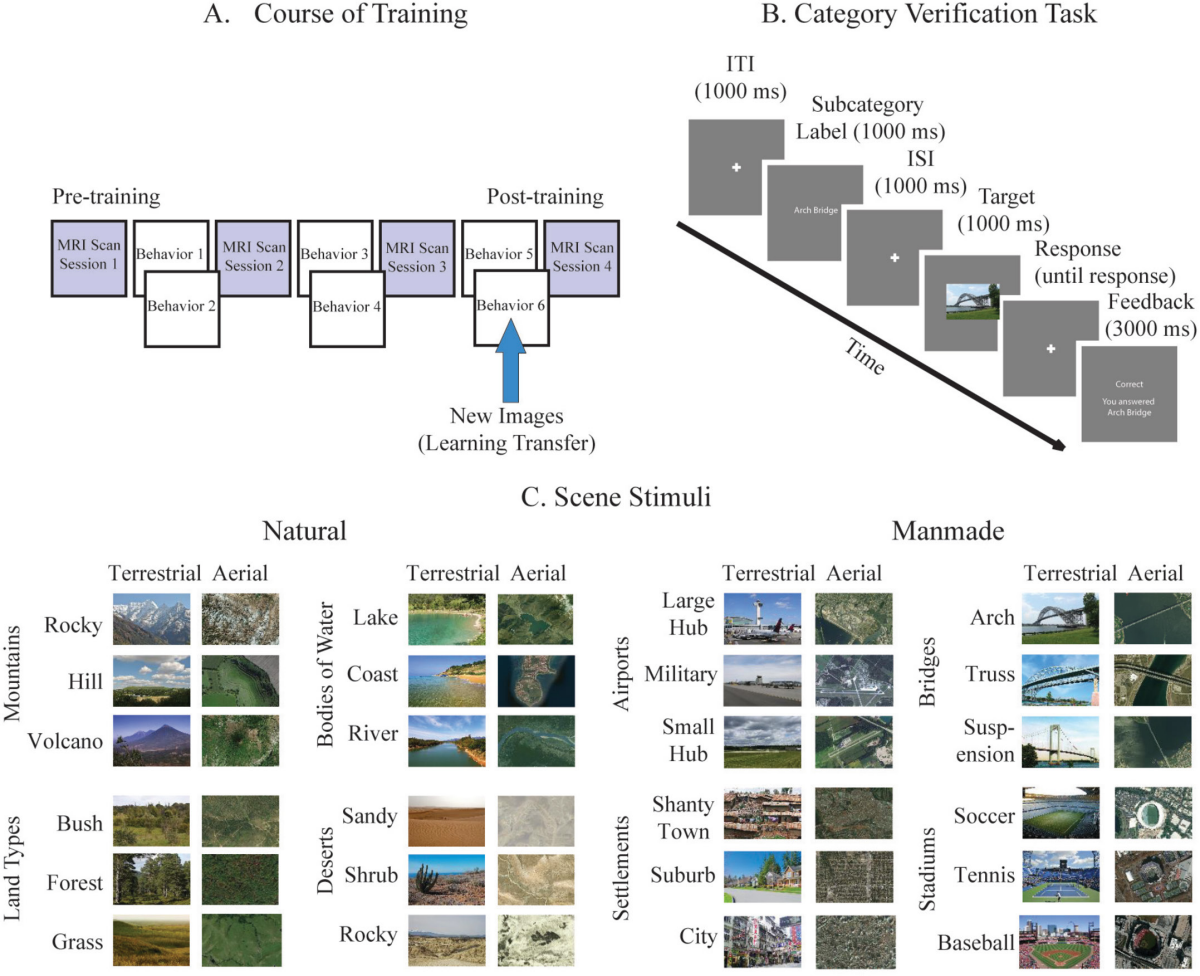
Experimental design and procedures. (A) Course of training. (B) The paradigm used was a category verification paradigm. (C) Examples of scene stimuli used in the study and their category labels. Each image depicts the same place from a terrestrial and aerial viewpoint (depicted here are one of 10 exemplars for each category). The images span both natural and man‐made categories, four categories within each (Natural: Mountains, Land Types, Bodies of Water, and Deserts; Man‐made: Airports, Settlements, Bridges, and Stadiums). Each subcategory contained three subordinate categories as labeled here (labels were not presented during the neuroimaging sessions).

Both the man‐made and natural scenes comprised four basic‐level categories: The man‐made scenes used were airports, bridges, stadiums, and settlements, whereas the natural scenes were mountains, deserts, land types, and bodies of water. Each of these basic‐level categories contained three subcategories and 10 exemplars within each subcategory. For example, the category “Deserts” contained three types of deserts: Sandy, Shrub, and Rocky, and each of these types of desert contained 10 individual images of specific desert landscapes (Figure 1A).

### 2.3 Experimental Design and Procedures

A full‐training regimen consisted of six behavioral training sessions interleaved with four MRI scanning sessions (for the full‐training design, see Figure 1B). Critically, each subject was exposed to a unique set of stimuli. A unique set of 240 individual scene stimuli was generated for each individual (both in the experimental and the control group), by randomly selecting five of the 10 exemplars in each subcategory while maintaining the scene dimensional hierarchy depicted above (Figure 1A). The first five behavioral sessions used the same individually picked 240 images, allowing us to test the formation of the subject's recognition memory (we refer to this as *within‐set learning*). The sixth session consisted of the other 240 scenes, that is, the scenes that the subject had not seen in the prior five sessions. This allowed us to test the subject's learning transfer (i.e., their ability to *generalize* what they learned during the within‐set learning to novel instances of the same scene categories). In addition to the behavioral training sessions, subjects were scanned four times: pre‐training (baseline), two scans during the course of training, and posttraining. Notably, subjects saw the same scene images in the fMRI scanner that they trained on during the first five behavioral training sessions.

In the experimental group, subjects were trained to categorize the scenes at the subcategory level using a category verification paradigm (Harel and Bentin 2009). Each trial began with the presentation of a subcategory label (e.g., “sandy desert”), followed by a scene image. The subject's task was to determine whether the image fits the category scene label or not (feedback was provided at the end of each trial) (Figure 1C). Every image was presented three times during the session, culminating in 720 individual trials per training session. The control group, in contrast, was exposed to the same scene images for the same number of times but performed an orthogonal fixation‐cross task, which did not require them to actively categorize the scenes (see Text S1 for more information on the control group behavioral task).

Subjects in both groups viewed the stimuli on a “24” LCD monitor at a viewing distance of approximately 57 cm. Each image was 600 × 400 pixels in size (subtending 15.88°× 10.52° visual angle). All stimuli were displayed at the center of the screen on top of a gray background.

### 2.4 Behavioral Analysis

Mean reaction times and accuracy scores were calculated for each condition. Any outliers were replaced with the subjects' overall mean across conditions within‐session. Inverse Efficiency Scores (IES) were calculated to accommodate for any speed‐accuracy trade‐off. IES was calculated by taking mean response time divided by the proportion correct (Bruyer and Brysbaert 2011). The individual IES means were then submitted to ANOVAs assessing two metrics of performance: *within‐set learning* (i.e., recognition memory for the same images across the five sessions), (2) *generalization* to new exemplars (i.e., learning transfer, considered a hallmark of perceptual expertise). For RT and Accuracy behavioral performance analyses for the experimental group see Text S1 and Figure S2.

### 2.5 fMRI Procedures

In the scanner, subjects from both groups performed a 1‐back task. This task was used because it requires only minimal knowledge of/or engagement with the scene images, and thus allows us to compare the two groups while controlling the subjects' overall engagement and experience (a common caveat in many studies of visual expertise, see Harel et al. 2010). We employed a typical block‐design fMRI experiment with Naturalness and Viewpoint as independent variables, yielding four conditions: man‐made‐terrestrial, man‐made‐natural, natural‐terrestrial, and natural‐aerial. A single scan session consisted of four experimental runs: all 240 images were presented within a single run. A run comprised 20 blocks, each of the four conditions presented in five blocks, pseudorandomized within a session across subjects. Each block consisted of 12 individual scene images (+2 repetitions for the 1‐back task), spanning the 60 individual scenes/conditions. Blank (fixation) blocks were included and interspersed between experimental blocks. The duration of experimental blocks was seven TRs (14 s), and the blank blocks were four TRs long (8 s). Individual stimuli within an experimental block were presented for 500 ms, followed by a 500‐ms ISI (gray screen).

In addition, each subject completed a block‐design category localizer scan to identify scene‐selective regions (PPA and OPA), object‐selective cortex (LOC), and nonselective, EVC. Although the retrosplenial complex (RSC) is also relevant for scene processing, we were unable to reliably collect ROI data across participants due to poor signal quality observed across sessions. Given these limitations, we did not include RSC in our final analyses. This fMRI experiment utilized a block design with four stimulus conditions: faces, houses, objects, and simple textures. Each condition was presented seven times in a pseudorandom order, and each block lasted 9 s followed by a 6‐s fixation period. Within each block, participants viewed nine images from the same category, with each image displayed for 800 ms, followed by a 200‐ms blank screen. All stimuli were grayscale photographs (300 × 300 pixels) and subtended a visual angle of 12° × 12°. Participants performed a standard one‐back memory task without overt responses, with image repetitions occurring once or twice per block (Harel et al. 2010). ROIs were generated from these maps by taking the contiguous clusters of voxels that exceeded threshold and occupied the appropriate anatomical location based on previous work.

### 2.6 Magnetic Resonance Imaging Parameters

Subjects were scanned in a 3‐T GE MRI (Discovery 750w, GE Healthcare, Madison, WI) using a 24‐channel head coil. We obtained the BOLD contrast with gradient‐echo echo‐planar imaging (EPI) sequence: time repetition (TR) = 2 s, time echo (TE) = 24 ms, flip angle = 90°, field of view (FOV) 24 × 24 cm, and matrix size 96 × 96 (in‐plane resolution of 3 × 3 mm). Each scan volume consisted of 27 axial slices of 3 mm thickness and 0.5‐mm gap covering the majority of the cortex. High‐resolution (1.1 × 1.1 mm) T1‐weighted anatomic images of the same orientation and thickness as the EPI slices were also acquired in each scan session to facilitate the incorporation of the functional data into the 3D Talairach space.

### 2.7 fMRI Preprocessing

We used BrainVoyager software package (Brain Innovation, Maastricht, the Netherlands) to analyze the fMRI data. The first three images of each functional scan were removed. A standard preprocessing pipeline was then applied to the functional scans consisting of 3D motion correction, slice scan time correction, spatial smoothing (FWHM = 5 mm), linear trend removal, and low‐frequency filtering. The preprocessed functional time series were aligned to high‐resolution (1.1 × 1.1 mm) T1‐weighted anatomic images of the same orientation and thickness using header‐based coregistration. The coregistered data were normalized into Talairach space (Talairach and Tournoux 1988).

### 2.8 fMRI ROI Analysis

ROIs were identified in each subject separately based on the category localizer experiment as described above. They were defined on the basis of a minimum cluster size of six contiguous functional voxels that exhibited selective activations in response to a specific category (e.g., houses > faces; *p* < 0.05). PPA ROIs were defined as regions residing in the parahippocampal gyrus (PHG) or the adjacent collateral sulcus (CoS) that showed a preferential activation to houses relative to faces. OPA ROIs were defined as regions residing in the vicinity of the transverse occipital sulcus (TOS) that showed a preferential activation to houses relative to faces. LOC ROIs were collectively defined as the combination of voxels in the lateral occipital aspect of the cortex in the vicinity of the inferior occipital sulcus or gyrus (LO) as well as in the posterior Fusiform sulcus (pFS) that showed a preferential activation to objects relative to textures (Grill‐Spector et al. 2000). Early visual areas were defined as regions in striate and extrastriate cortex in the medial aspect of the cortex that showed a preferential activation to textures relative to objects (Harel et al. 2010). ROIs were of comparable size and did not vary across the two groups (see Table S1) and subsequently no additional normalization or size equation procedures were applied to the data.

We sampled the time courses of activation in the main experiment in each ROI, computing the percent of BOLD signal change compared with the fixation period preceding it. Put simply, for each participant, in each independently selected ROI, we have extracted the average time courses (across blocks) for each run for each of the four experimental conditions. Percent signal change (PSC) was then computed using BrainVoyager software by comparing each time point of that time course signal with a baseline signal (a pre‐specified baseline period of two TRs prestimulus onset) and then normalized and converted to a percentage value (see also: time course normalization). Having extracted the PSC for all voxels within a given ROI (left and right ROIs were extracted separately), these were then averaged across all voxels and exported for offline analysis outside of BrainVoyager. In this offline analysis, we averaged across runs and extracted (for each ROI in each hemisphere) the average response (PSC averaged across a uniform 4‐TR time window) for each of the four experimental conditions.

Because no hemispheric differences were found for any of the ROIs, the right and left hemisphere ROI time courses were combined by a weighted average. Finally, for each ROI and for each condition, the time courses were averaged for each participant of each group. These were then subjected to a factorial ANOVA with Viewpoint (aerial and terrestrial), Naturalness (man‐made and natural), and Session (first, second, third, and fourth) as within‐subject factors and Group (experimental and control) as a between‐subject factor. Simple effects analyses following significant interactions comprised post hoc comparisons to make specific pairwise comparisons within each level of the other factor. All post hoc comparisons reported here are Bonferroni corrected for multiple comparisons. Note that parts of the current work were presented in an exploratory study (Borders et al. 2020). However, the previous work was not focused on the question of training in scene categorization and its uniqueness to specific regions in OTC and did not include ROI analyses.

### 2.9 Brain–Behavior Correlation Analyses

To correlate between behavioral metrics of training efficacy and underlying neural activity, we derived two general behavioral indices: (1) a within‐set learning index and (2) a generalization index, and correlated each of these measures with the mean response magnitude in each of the four ROIs. The *within‐set learning index* was calculated as the ratio between performance levels in last and first behavioral sessions using the same identical stimuli (i.e., Session 5/Session 1). Thus, if there were no improvement over the course of training, that ratio would equal 1, and the greater the improvement over time (lower IES), the lower the ratio. Conversely, the *generalization index* was calculated as the ratio between performance levels in two different behavioral sessions: between the sixth and the second behavioral sessions (i.e., Session 6/Session 2). If transfer of learning indeed occurs then performance with novel, never seen‐before images should be at least as good as performance after a single session of training. Thus, a ratio of 1 would mean equivalent level performance in the two sessions, meaning participants were able to generalize what they learned from two training sessions (i.e., applying the same scene categorization scheme), to novel instances of these stimuli. Values smaller than 1 would indicate an even greater learning transfer, whereas values above 1 would indicate no substantial generalization occurred. We calculated the two indices across the two scene properties (viewpoint and naturalness) and performed Pearson's correlation analysis between each of the indices and the mean response in each ROI across the four scanning sessions Statistical threshold for the brain–behavior correlations was set at *p* < 0.05. In order to compare the strength of the behavioral–brain correlations across the ROIs we conducted the Williams–Hotelling test (Williams 1959), a statistical test to compare two dependent correlation coefficients that share a common variable (in our case the behavioral metrics) from a single sample to determine if they differ.

## 3 Results

### 3.1 Behavioral Analyses

**FIGURE 2:**
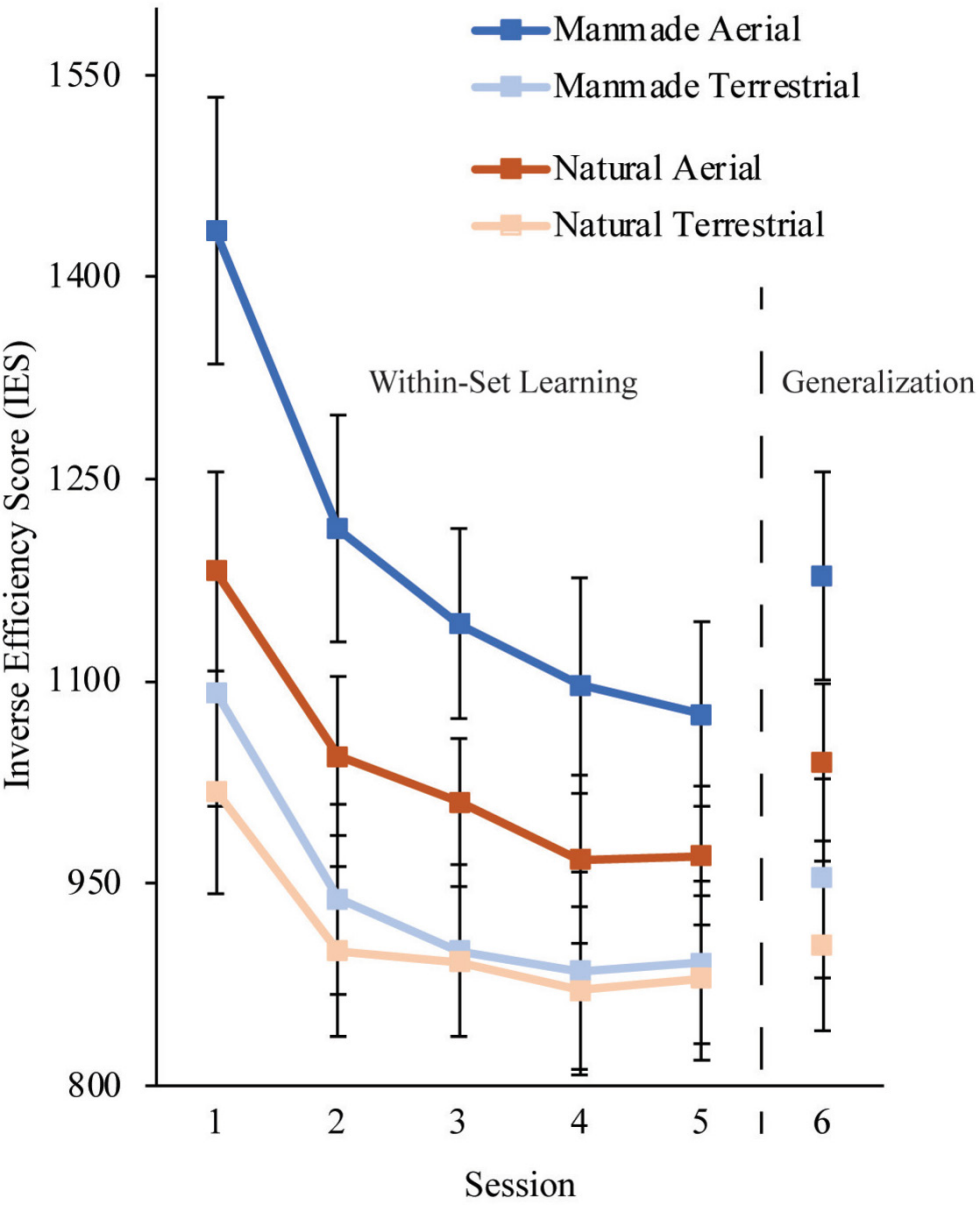
Learning trajectories across training sessions as a function of Naturalness and Viewpoint.

To measure the efficiency of the categorization training regimen, we assessed both within‐set learning (learning across the first five sessions) and generalization of learning (learning transfer) in the experimental group. Starting with within‐set learning, we conducted a repeated‐measures ANOVA on participants' categorization performance scores, measured by inverse efficiency scores (IES) using Naturalness (man‐made and natural), Viewpoint (aerial and terrestrial), and Session (1–5) as independent variables. We found learning effects over the course of the five training sessions (main effect of Session: *F*(4,68) = 14.02, *p* < 0.001, *η*_*p*_^2^ = 0.452). Categorization performance improved with each subsequent session, as indicated by a significant linear decrease (*F*(1,17) = 19.651, *p* < 0.001, *η*_*p*_^2^ = 0.536), with the most drastic learning occurring between the first (*M* = 1182 ms, SEM = 80) and second (*M* = 1024 ms, SEM = 67) training sessions (*t*(17) = 4.57, *p* < 0.001) (Figure 2).

In addition, we found that overall, categorization performance varied as a function of scene properties. Terrestrial scenes (*M* = 927 ms, SEM = 56) were categorized more efficiently than aerial scenes (*M* = 1114 ms, SEM = 71) (main effect of Viewpoint: *F*(1,17) = 65.197, *p* < 0.001, *η*_*p*_^2^ = 0.793). Natural scenes (*M* = 973 ms, SEM = 62) were categorized better than man‐made scenes (*M* = 1067 ms, SEM = 74) (main effect of Naturalness: *F*(1,17) = 41.76, *p* < 0.001, *η*_*p*_^2^ = 0.711). Moreover, the two scene properties had an interactive effect on performance (Viewpoint‐by‐Naturalness interaction: *F*(1,17) = 34.4, *p* < 0.001, *η*_*p*_^2^ = 0.67); the difference between aerial and terrestrial views was more pronounced in the man‐made scenes (Man‐made Aerial: *M* = 1193 ms, SEM = 77; Man‐made Terrestrial: *M* = 941 ms, SEM = 54; *t*(17) = 7.38, *p* < 0.001) compared to the natural scenes (Natural‐Aerial: *M* = 1035 ms, SEM = 66; Natural‐Terrestrial: *M* = 912 ms, SEM = 59; *t*(17) = 7.66, *p* < 0.001) (Figure 2).

**FIGURE 3:**
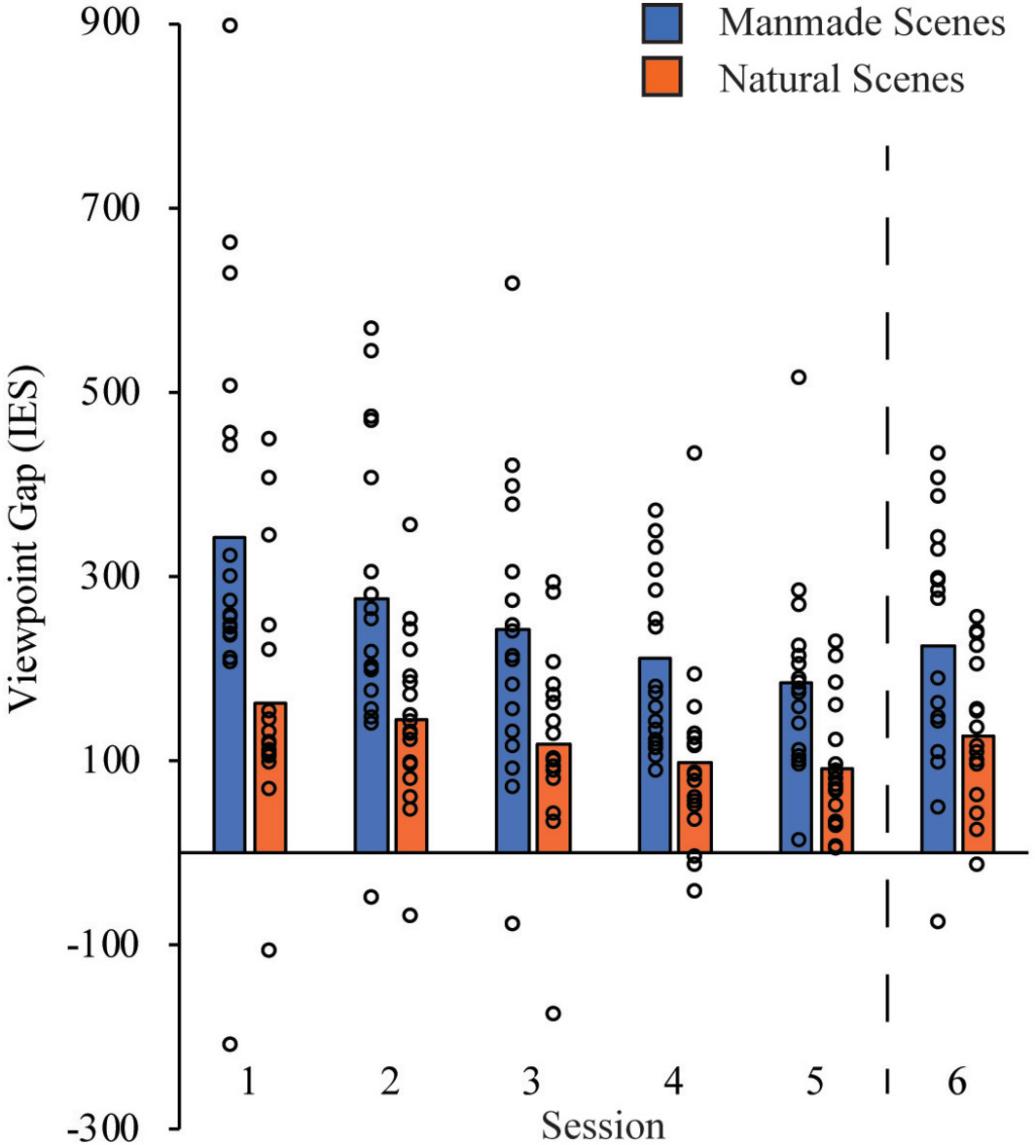
Viewpoint gap by Naturalness across training sessions.

Lastly, the impact of the scene properties on categorization performance varied across the training sessions (Viewpoint‐by‐Naturalness‐by‐Session interaction: *F*(4,68) = 2.869, *p* < 0.029, *η*_*p*_^2^ = 0.144). Categorizing scenes from the two unique viewpoints (aerial vs. terrestrial) as belonging to the same group (i.e., subcategory) changed over the course of training based on whether the participants were categorizing the scenes as man‐made (Session main effect: *F*(4,68) = 8.42, *p* < 0.001, *η*_*p*_^2^ = 0.33) or natural (Session main effect: *F*(4,68) = 2.42, *p* < 0.06, *η*_*p*_^2^ = 0.12). Thus, with increasing training, the performance gap between aerial and terrestrial viewpoints of the same places was minimized (Figure 3).

Next, we examined the extent of learning transfer achieved by the participants. First, we compared Session 1 (categorizing novel scenes for the first time) with Session 6 (categorizing novel scenes after having gone through 6 training sessions) and found that categorization of novel scenes after completing the training regimen was significantly better than categorization at the onset of the training (*F*(1,17) = 7.066, *p* = 0.017, *η*_*p*_^2^ = 0.294). Participants' categorization performance in Session 6 predictably varied as a function of the scene properties, relative to the earlier training sessions. Terrestrial scenes were categorized more efficiently than aerial scenes (*F*(1,17) = 49.98, *p* < 0.001, *η*_*p*_^2^ = 0.75), and man‐made scenes were categorized more readily than terrestrial scenes (*F*(1,17) = 61.15, *p* < 0.001, *η*_*p*_^2^ = 0.78). Second, we found that average performance across the training sessions (2–5) was equivalent to performance in the sixth session (*F*(1,17) = 1.811, *p* = 0.196, *η*_*p*_^2^ = 0.096; *BF*₁₀ = 0.21, *BF*₀₁ = 4.65), which suggests that participants categorized new scenes equally well as they did with the familiar scenes they were trained with. For an analysis of the behavioral performance of the control group, see Text S1 and Figure S1.

### 3.2 ROI Analysis

To assess how experience and training with novel scenes influence category‐selective regions in OTC, we analyzed response modulation in scene‐selective areas (PPA and OPA), an object‐selective region (LOC), and a general‐purpose visual area (EVC). We examined how response magnitude varied by region, as well as the following experimental factors: Sessions (1, 2, 3, and 4), Naturalness (man‐made and naturalness), and Viewpoint (aerial and terrestrial). We present our findings in the following order: First, we asked how scene representations change over time, with repeated exposure (main effect of Session and other interactions with Session). Second, we examined how the different ROIs respond to the two key scene properties we manipulated, looking at sensitivity to aerial and terrestrial scenes (main effect of Viewpoint) and to natural and man‐made scenes (main effect of Naturalness). Third, we investigated how acquiring semantic knowledge over time by training in scene categorization impacts existing perceptual representations both within and outside scene‐selective cortex. Specifically, we examined whether such putative modulation is unique to the training regime or whether it reflects mere exposure to the learned scenes (Group interactions).

Prior to examining these three questions, we note we observed a significant main effect of ROI (*F*(3,111) = 73.346, *p* < 0.001, *η*_*p*_^2^ = 0.665), with EVC showing a stronger response (*M* = 0.635, SEM = 0.039) compared to LOC (*M* = 0.202, SEM = 0.02), OPA (*M* = 0.486, SEM = 0.026), and PPA (*M* = 0.316, SEM = 0.016) (post hoc pairwise comparisons, all *p*s < 0.001). We refer the reader to Table S2 for the full list of all statistical analyses.

#### 3.2.1 General Learning Effects

**FIGURE 4:**
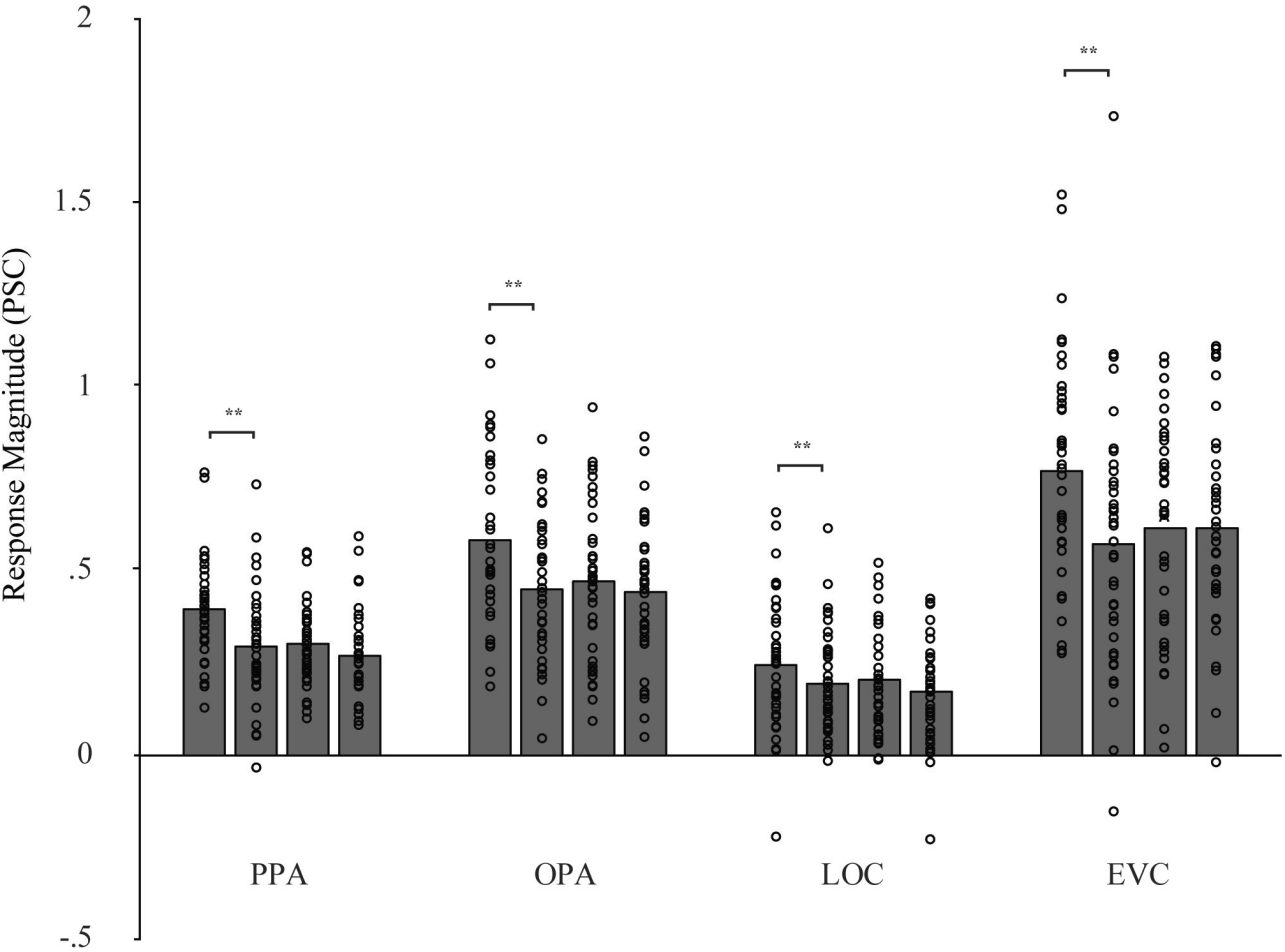
The effect of session on the response magnitude (in percent signal change, PSC) of the four ROIs studied. *p < 0.05; **p < 0.01.

To test the effect of repeated exposure on response magnitude over time, we conducted a four‐way ANOVA with ROI (PPA, OPA, LOC, and EVC), Sessions (1, 2, 3, and 4), Viewpoint (aerial, terrestrial), and Naturalness (man‐made, natural) as independent factors with response magnitude as a dependent measure. Note that since our focus is on the overall general effects of repeated exposure to scenes, we averaged the effects across the experimental and control group (Figure 4). We observed a main effect of Session (*F*(3,111) = 11.577, *p* < 0.001, *η*_*p*_^2^ = 0.238), with the largest response magnitude decrease between the first (*M* = 0.497, SEM = 0.027) and second (*M* = 0.377, SEM = 0.026) sessions (*p* < 0.005). There were no additional differences between successive levels of Session. Slight variations between ROIs were observed, manifested in a significant ROI‐by‐Session interaction (*F*(9,333) = 2.905, *p* < 0.005, *η*_*p*_^2^ = 0.073). Subsequent analyses revealed that all ROIs showed a significant Session effect (all *p*s < 0.05), with a significant difference between Sessions 1 and 2 (*p* < 0.05). However, this difference did not reach significance in LOC (*p* = 0.08).

#### 3.2.2 Sensitivity to Scene Properties (Naturalness and Viewpoint)

**FIGURE 5:**
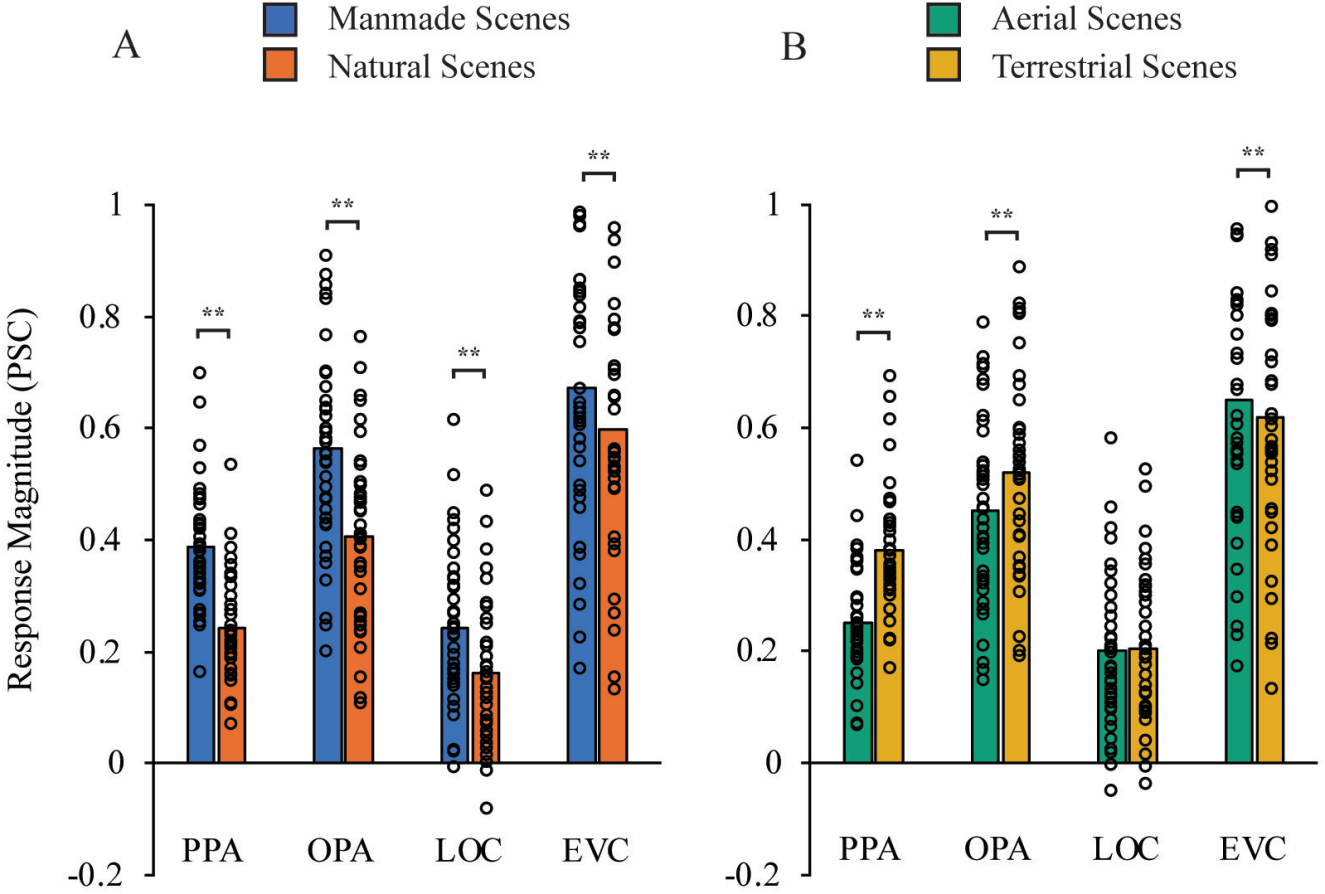
The effects of scene properties on response magnitude. (a) The effect of Naturalness on response magnitude (PSC) for each of the four ROIs. (b) The effect of Viewpoint on response magnitude (PSC) in each of the four ROIs. *p < 0.05; ** *p* < 0.01.

We observed a significant main effect of Naturalness (*F*(1,37) = 700.928, *p* < 0.001, *η*_*p*_^2^ = 0.950), with man‐made scenes (*M* = 0.47, SEM = 0.02) eliciting greater neural activity than natural scenes (*M* = 0.35, SEM = 0.02). All regions exhibited a Naturalness effect, with pronounced differences across regions (ROI‐by‐Naturalness interaction: *F*(3,111) = 51.184, *p* < 0.001, *η*_*p*_^2^ = 0.580; Figure 5A). The Naturalness effect (operationalized as the difference score between man‐made and natural scenes) was more robust in scene‐selective regions (PPA and OPA) than in nonscene‐selective regions (both EVC and LOC) (Main effect of ROI: *F*(3,114) = 51.121, *p* < 0.001, *η*_*p*_^2^ = 0.574). PPA and OPA both displayed a comparable Naturalness effect (*M* = 0.145, SEM = 0.006; *M* = 0.16, SEM = 0.008, respectively), which was significantly stronger than in either LOC (*M* = 0.082, SEM = 0.006, *p*s < 0.001) or EVC (*M* = 0.076, SEM = 0.007, *p*s < 0.001).

Another aspect of the difference between scene‐selective and nonscene‐selective regions in their response to scene naturalness was how the Naturalness effect changed over time. We observed a significant ROI‐by‐Session‐by‐Naturalness interaction (*F*(9,333) = 2.771, *p* < 0.005, *η*_*p*_^2^ = 0.957). Examining how the Naturalness effect in each region changed with increasing experience, we found that although in both LOC and EVC the Naturalness effect stayed constant across sessions (LOC: *F*(3,114) = 2.576. *p* < 0.06, *η*_*p*_^2^ = 0.063; EVC: *F*(3,114) < 1.00, *η*_*p*_^2^ = 0.024), in contrast, both PPA and OPA showed significant effects of Session on the Naturalness effect (PPA: *F*(3,114) = 7.833. *p* < 0.001, *η*_*p*_^2^ = 0.171; OPA: *F*(3,114) = 5.615. *p* < 0.005, *η*_*p*_^2^ = 0.129).

The other scene property we manipulated in the study was Viewpoint. We observed a significant main effect of Viewpoint (*F*(1, 37) = 95.958, *p* < 0.001, *η*_*p*_^2^ = 0.722), with terrestrial scenes (*M* = 0.432, SEM = 0.019) eliciting greater activation compared to aerial scenes (*M* = 0.388, SEM = 0.019). This Viewpoint effect, operationalized as the difference score between terrestrial and aerial scenes, was more pronounced in scene‐selective regions (PPA and OPA) than in nonscene‐selective regions (both EVC and LOC; Figure 5B). The main effect of ROI was significant, with the Viewpoint difference as the dependent measure (*F*(3,114) = 90.104, *p* < 0.001, *η*_*p*_^2^ = 0.703). PPA exhibited the most robust Viewpoint effect (*M* = 0.133, SEM = 0.011), followed by OPA (*M* = 0.069, SEM = 0.008). Both regions showed significantly stronger effects (with greater activation for terrestrial relative to aerial scenes) compared to LOC (*M* = 0.005, SEM = 0.006, *p*s < 0.001) and EVC (*M* = −0.029, SEM = 0.006, *p*s < 0.001) (Figure 5B).

**FIGURE 6:**
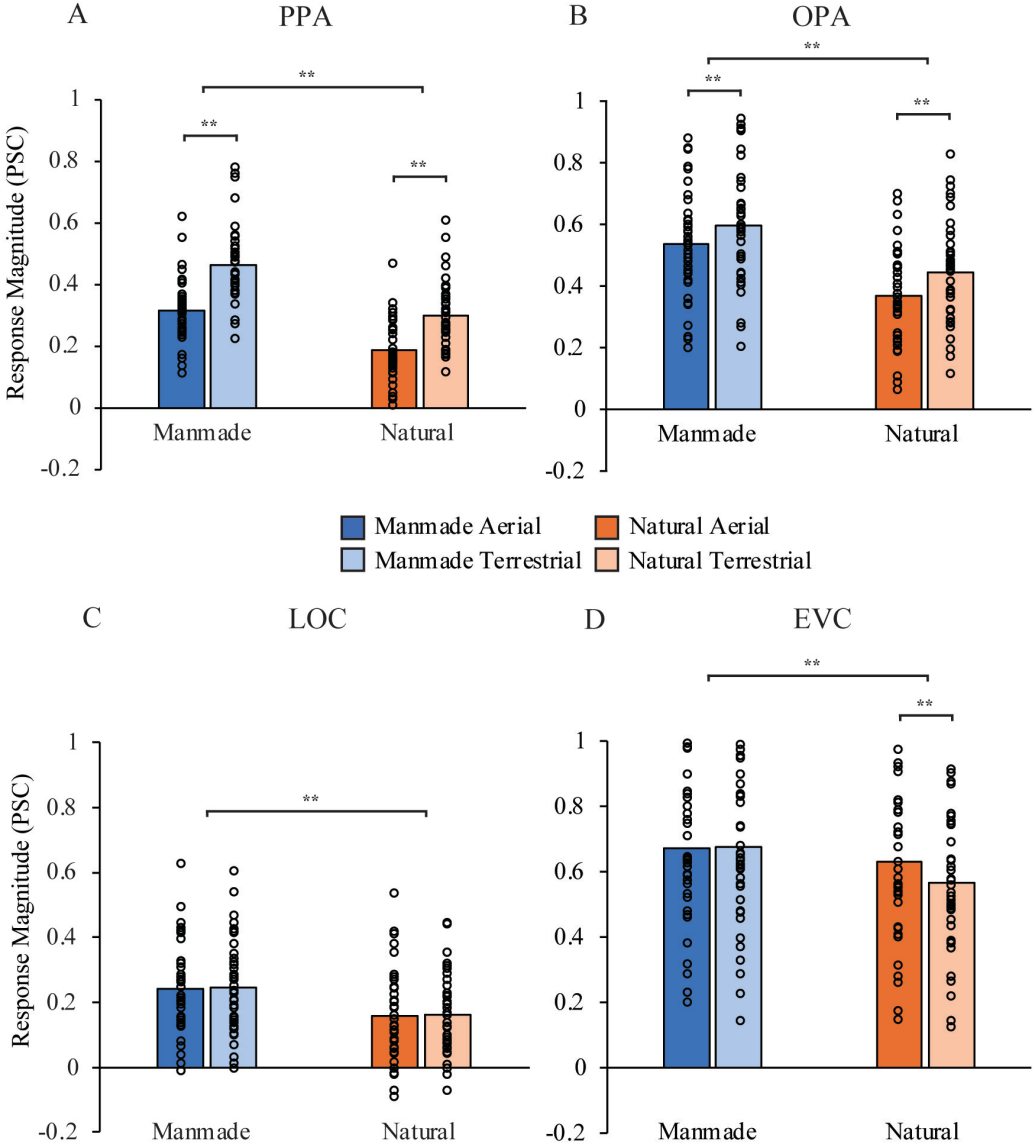
The joint effects of Naturalness and Viewpoint on response magnitude (PSC) in each of the four ROIs. Top row: scene‐selective regions (PPA and OPA). Bottom row: Control regions (LOC and EVC). *p < 0.05; **p < 0.01.

The two scene properties showed a combined effect on response magnitude (Naturalness‐by‐Viewpoint interaction: *F*(1,37) = 14.507, *p* < 0.001, *η*_*p*_^2^ = 0.282), which manifested differently across the ROIs (Naturalness‐by‐Viewpoint‐by‐ROI interaction: *F*(3,111) = 28.465, *p* < 0.001, *η*_*p*_^2^ = 0.435). To further pursue this interaction, we examined how the viewpoint index varied as a function of Naturalness in each one of the ROIs. PPA exhibited a positive viewpoint effect that was stronger for the man‐made scenes (*M* = 0.153, SEM = 0.013) compared to natural scenes (*M* = 0.114, SEM = 0.011) (*p* < 0.001). OPA also showed a positive Viewpoint effect, albeit in the opposite direction: A more pronounced effect was observed for natural scenes (*M* = 0.079, SEM = 0.009) relative to man‐made scenes (*M* = 0.061, SEM = 0.009), (*p* < 0.02). Note that all viewpoint indices were significantly greater than 0 (all *p*s < 0.001) (Figure 6).

EVC also demonstrated significant differences in the Viewpoint effect between natural and man‐made scenes, although with a negative viewpoint effect (aerial scenes exhibited a greater activity than terrestrial scenes) that was more pronounced for natural scenes (*M* = −0.064, SEM = 0.008) compared to man‐made scenes (*M* = 0.004, SEM = 0.008) (*p* < 0.001). Indeed, whereas the viewpoint effect for natural scenes was significantly different from 0 (*p* < 0.001), the viewpoint effect in the man‐made scenes was not significantly different from 0 (*p* > 0.55). Lastly, LOC showed no significant combined effect of Naturalness and Viewpoint (*F*(1,38) < 1.00, *η*_*p*_^2^ < 0.001).

#### 3.2.3 The Impact of Categorization Training on Response Magnitude

To establish whether the impact of training in scene categorization is specific to scene‐selective cortex, we examined how differences in response magnitude between the experimental and control groups evolved across training sessions. We investigated all interactions involving ROI, Group, Session, and the two additional scene properties. This resulted in a complex pattern of interactions that underscores the nuanced effects of the training paradigm.

**FIGURE 7:**
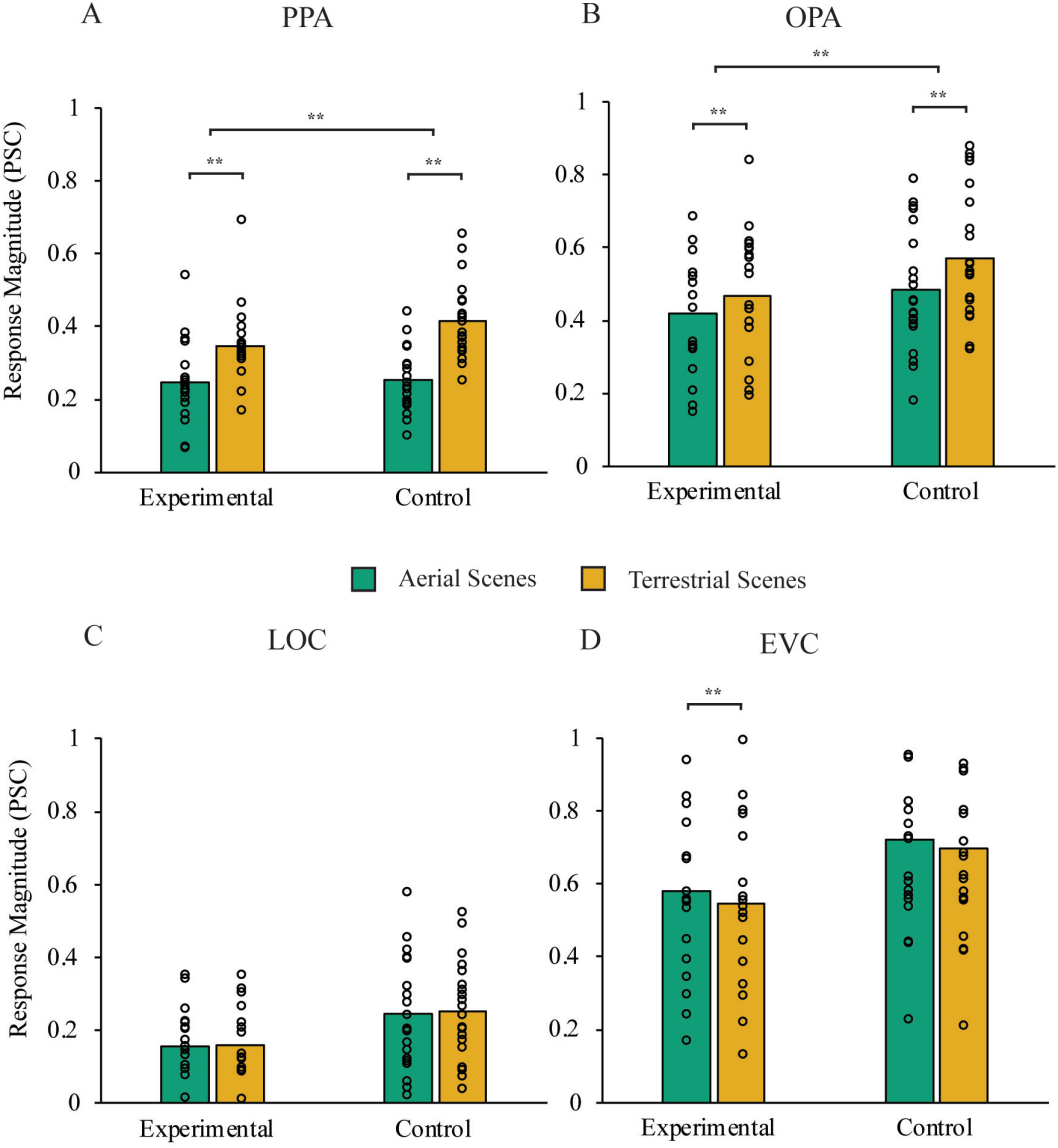
The joint effects of Viewpoint and Group on response magnitude (PSC) in each of the four ROIs. Top row: scene‐selective regions (PPA and OPA). Bottom row: Control regions (LOC and EVC). *p < 0.05; **p < 0.01.

First, we found that training (in the form of a group interaction, albeit across sessions) impacts how participants extract viewpoint information (ROI‐by‐Viewpoint‐by‐Group interaction: *F*(3,111) = 3.176, *p* < 0.03, *η*_*p*_^2^ = 0.079). Follow‐up analyses of this interaction revealed the effect was particularly conspicuous in scene‐selective cortex (Figure 7). Specifically, we found that both PPA (*M*_experimental_ = 0.10, SEM = 0.015; *M*_control_ = 0.16, SEM = 0.014) and OPA (*M*_experimental_ = 0.05, SEM = 0.011; *M*_control_ = 0.08, SEM = 0.011) showed a significant effect of Group (*p* < 0.005*; p* < 0.03, respectively) on the viewpoint gap (see above), with both regions showing a smaller gap in the experimentals compared to the controls. In contrast, neither LOC (*M*_experimental_ = 0.003, SEM = 0.009; *M*_control_ = 0.007, SEM = 0.008) nor EVC (*M*_experimental_ = −0.035, SEM = 0.009; *M*_control_ = −0.024, SEM = 0.008) showed any significant effects of Group on the viewpoint gap (*p* > 0.70; *p* > 0.35, respectively). This finding suggests that training modulates the processing of viewpoint information specifically in scene‐selective cortex.

**FIGURE 8:**
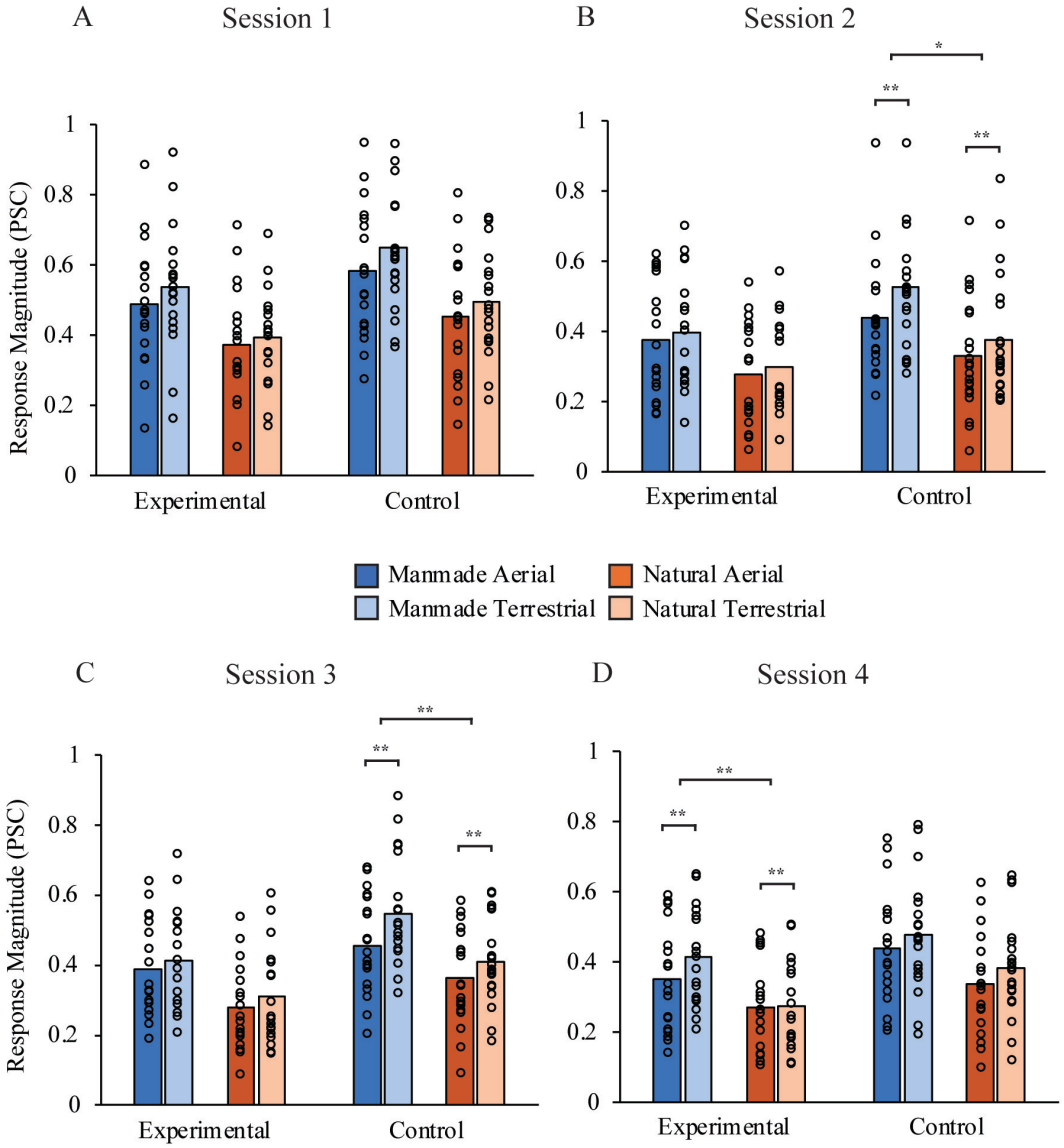
The joint effects of Naturalness, Viewpoint, Session, and Group on response magnitude (PSC) collapsed across all ROIs. *p < 0.05; **p < 0.01.

Interestingly, the above training effect did not extend to higher order interactions involving ROI and Session (ROI‐by‐Session‐by‐Naturalness‐by‐Viewpoint‐by‐Group interaction: *F*(9,333) < 1.00, *η*_*p*_^2^ = 0.02; ROI‐by‐Group‐by‐Session interaction: *F*(9,333) > 1.00, *η*_*p*_^2^ = 0.011), limiting our ability to establish how early over the course of training the scene‐specific group effect formed. Although not ROI‐specific, another interesting aspect of the training was manifested in a significant four‐way interaction between Session, Group, Naturalness, and Viewpoint (*F*(3,111) = 6.635, *p* < 0.005, *η*_*p*_^2^ = 0.268). We conducted follow‐up analyses, examining how the viewpoint gap differs across the man‐made and natural scenes in each of the two groups over the different training sessions (Figure 8; note that the Aerial and Terrestrial conditions are presented separately). Starting with the experimental group, we found that in the first three sessions, the viewpoint gap did not differ significantly between the man‐made and natural scenes (all *p*s > 0.2), whereas in Session 4 the Viewpoint gap was more pronounced in the man‐made scenes compared to the natural scenes (*M*_manmade_ = 0.061, SEM = 0.017; *M*_natural_ = 0.003, SEM = 0.012) (*p* < 0.005). In the control group, the Viewpoint‐by‐Naturalness interaction manifested differently over the course of training. Differences across levels of Naturalness were evident in Session 2 (*M*_manmade_ = 0.089, SEM = 0.018; *M*_natural_ = 0.047, SEM = 0.014) (*p* < 0.02) and Session 3 (*M*_manmade_ = 0.090, SEM = 0.012; *M*_natural_ = 0.046, SEM = 0.01) (*p* < 0.005) but not in Sessions 1 and 4 (*p* > 0.20, *p* > 0.55, respectively). Although somewhat nonintuitive, the results nonetheless indicate a differential utilization of scene properties information over time between the two groups.

To summarize our ROI results, we found that scene‐selective cortex (PPA and OPA) and control regions (LOC and EVC) show a reduction in activation with repeated exposure. However, scene‐selective regions differed from the control regions: They showed a stronger response for terrestrial compared to aerial scenes, whereas EVC showed the opposite pattern and LOC was indifferent to viewpoint information. Further, the effect of training in scene categorization on the utilization of viewpoint information was relegated to scene‐selective cortex and did not manifest in the control regions.

### 3.3 Brain–Behavior Correlations

Next, we sought to establish which of the ROIs were most strongly correlated with the behavioral training effects. We hypothesized that the level of activity should be predictive of training success (in the form of learning transfer) and that this correlation would be specific to scene‐selective cortex. To test this relationship, we correlated the generalization index (see Section 2 for details) with the mean response magnitude of each of the four ROIs in the experimental group. We also correlated mean activity in each of the ROIs with the within‐set learning index, as a measure of general learning.

**FIGURE 9:**
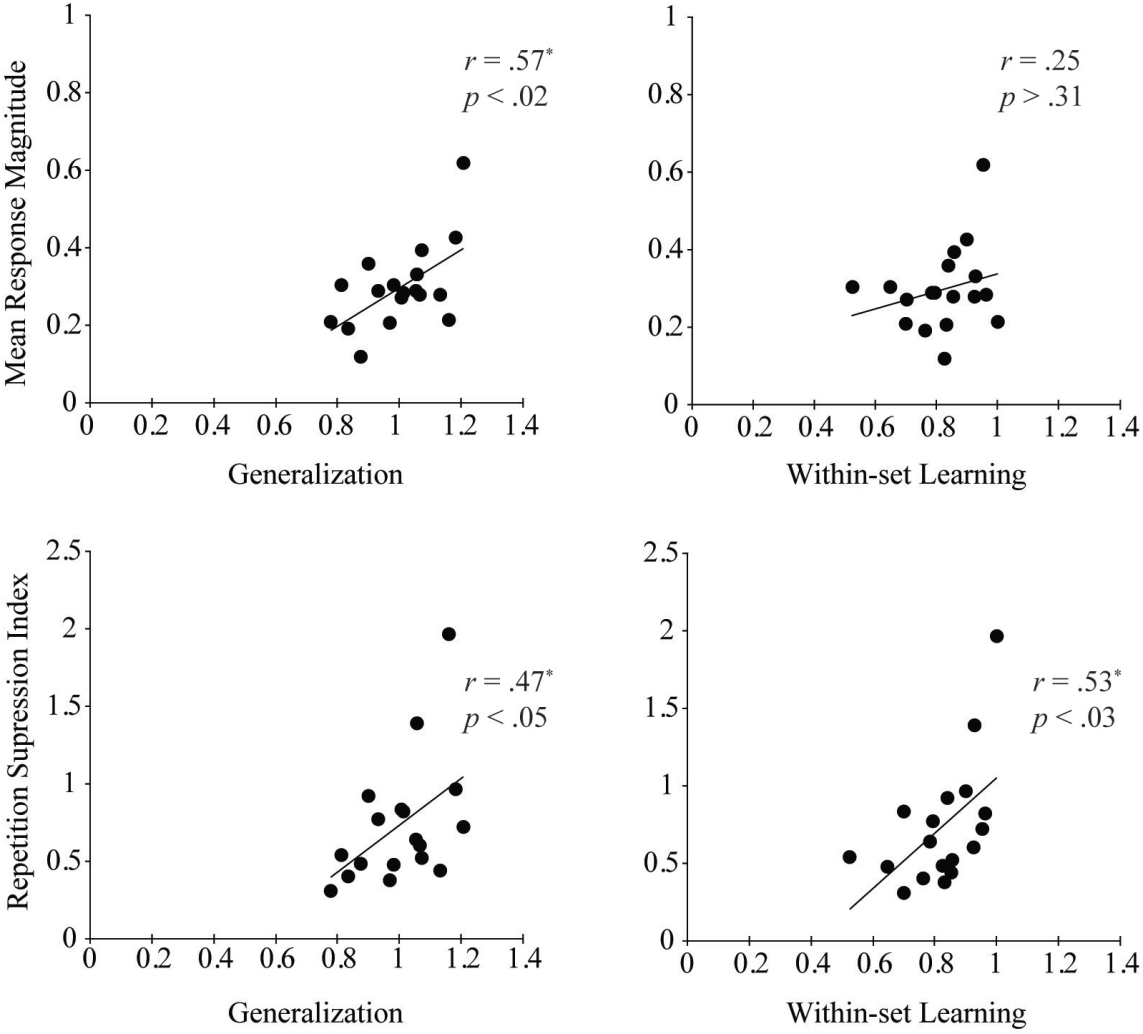
Correlations between the two behavioral performance metrics following training (Generalization: left column, Within‐set Learning: right column), and changes to PPA activity following training, presented as mean response (top row) and repetition suppression (bottom row). See main text for details. *p < 0.05.

We found that mean activity in PPA was significantly predictive of learning transfer (*r* = 0.57, *p* < 0.02). Further, mean activity in PPA was significantly correlated with improvement in posttraining performance, compared to both OPA and LOC (examined via Hotelling–Williams test for pairwise dependent correlations, *p* < 0.05) although it was not significantly different from EVC (*p* > 0.15). Note, however, that the correlation between mean activity in EVC and generalization was not significant (*r* = 0.16, *p* > 0.05 for full correlation results, see Table 1). Notably, mean activity in PPA was not significantly correlated with within‐set learning (*r* = 0.25, *p* > 0.30), and neither was mean activity in any of the other three ROIs (Figure 9; Table 1).

**TABLE 1:** Correlations between changes in brain activity and behavioral performance following training across regions of interest.

	Region of interest			
	PPA	OPA	LOC	EVC
Generalization				
*Mean response magnitude*	0.57*	0.21	0.12	0.16
*Repetition suppression*	0.47*	0.39	0.23	0.24
Within‐set learning				
*Mean response magnitude*	0.25	0.18	−0.09	0.14
*Repetition suppression*	0.53*	0.26	0.41	0.13

## 4 Discussion

Our study aimed to establish the impact of experience on scene‐selective cortex by studying how training in scene categorization impacts its activity relative to other occipitotemporal regions. We found that training in subordinate scene categorization presented from aerial and terrestrial viewpoints culminated in significant improvements to performance, both in recognition memory and learning transfer, two established hallmarks of expertise (Duyck et al. 2021; Wing et al. 2022). Neurally, we found that although all OTC regions decreased in response to repeated exposure to the same scenes, only scene‐selective cortex showed specific effects of training. The effect of experience (group by viewpoint interaction) was evident in both OPA and PPA. Further, PPA was the only region evincing significant posttraining neurobehavioral correlations with both recognition memory and learning transfer. These findings suggest that training‐induced modulation of neural activity is specific to scene‐selective cortex, particularly PPA, and provide novel evidence for the flexibility of scene‐selective cortex.

The effect of experience on scene recognition has received scant attention to date. The current work is among the few neuroimaging studies investigating how extended categorization training alters neural responses to scenes. Our findings that training to categorize scenes from an aerial perspective modulated activity primarily in scene‐selective cortex demonstrate the flexibility of the scene recognition system (Shelton and Gabrieli 2004; Shelton and McNamara 2004). We suggest that the changes in activity in scene‐selective cortex following training might underlie our ability to master new visual environments, environments our brains have not originally evolved to process (or at least are much less familiar with). Aerial scenes are, indeed, more difficult to categorize, as they lack vital holistic information inherent in terrestrial scenes (e.g., perceptual upright, see Loschky et al. 2015; Al Zoubi and Harel 2025). Behaviorally, we found that training categorization enables people to overcome the inherent difficulty of aerial scene categorization. Notably, this behavioral improvement is paralleled by a reduction in the response magnitude difference between aerial and terrestrial scenes in scene‐selective cortex in the trained group relative to controls. Therefore, we suggest that scene‐selective cortex supports the minimization of the “viewpoint gap,” reflecting the aerial scenes becoming “closer” (or rather, less unfamiliar) to the terrestrial scenes. We interpret this reduced “viewpoint gap” as reflecting generalization of PPA responses, suggesting one way by which existing terrestrial‐centric processes may be applied to novel aerial viewpoints (Barra et al. 2012; Ringer and Loschky 2018).

This account is preliminary as it is limited by the fact that we only assess response magnitude rather than response patterns (indicative of representational changes, see Harel et al. 2013a). However, support for this conjecture is provided by our finding that categorization training results in very specific changes: to scene‐selective cortex, training (group), and type of information (viewpoint). This implies that categorization training promotes more efficient utilization of viewpoint information by scene‐selective cortex. These changes in response to aerial scenes observed in scene‐selective cortex following training are quite distinct from changes in response magnitude in EVC (see Figure 6), suggesting that the effects cannot be fully explained by low‐level visual adaptation or general attentional gain.

All the ROIs decreased in their response magnitude irrespective of scene contents (viewpoint and naturalness), indicative of repetition suppression (RS: Gotts 2016). These RS effects were most pronounced between first and second scan sessions, consistent with previous research (Grill‐Spector et al. 2006). RS effects are often not content‐specific: Most category‐selective regions in OTC exhibit RS effects for stimuli from their nonpreferential category (Kim 2017; Lee et al. 2020). This contrasts with the specific nature of the training effects observed in our study. And although RS effects are pervasive in our study, it is important to distinguish the two mechanisms at play: visual learning and categorization training. Visual learning is a general perceptual phenomenon emerging from repeated exposure to the same stimuli, observed across multiple visual areas (Gotts 2016). Conversely, training in visual categorization requires active engagement rather than passive registration of the stimuli, a process culminating in higher level semantic representations modulating upstream perceptual processes (Iordan et al. 2024) and manifest as subsequent changes to representations in specialized cortical regions (Harel et al. 2016; Iordan et al. 2024). Although the two separate learning systems are independent, they do operate simultaneously, albeit at different magnitudes and spatial scales. Thus, repetition suppression produces a global, nonselective attenuation of signal across visual areas (consistent with widespread visual adaptation: Grill‐Spector et al. 2006), whereas scene categorization training yields smaller, yet functionally significant, category‐specific changes. The challenge for future studies of visual categorization training lies in teasing apart these two neural mechanisms while elucidating their respective temporal dynamics.

Several open questions still remain. First, are the observed changes in scene‐selective cortex dependent on the specific tasks used? We employed three behavioral paradigms: (1) a category verification paradigm, common in assessing training and expertise to train the experimental group (Scott et al. 2008); (2) a fixation‐cross task, which requires no explicit judgment of the scene, for the controls (Hansen et al. 2018); (3) a 1‐back task, which does not require prior knowledge of the scenes, demanding equal attentional engagement for both groups during scanning (see also Hansen et al. 2018). The 1‐back task, rather than category verification (the task participants trained on), was selected for scanning to avoid spurious group effects (resulting from differences in familiarity with the task rather than genuine differences in training). Given the diverse nature of the tasks, one could ask to what extent the training‐induced changes to PPA reflect the training paradigm, the 1‐back task, or their interaction? Given the difference between the behavioral training and scanning tasks, it might be possible that the neuroimaging results do not reflect the full impact of training. Even if that were the case, the fact that we still find training effects despite task differences points to their robustness. Demonstrating training effects across different tasks provides a more rigorous test of our hypothesis, as it offers evidence for cross‐task learning transfer.

Second, since the categorization task involved forming associations between certain viewpoints and their semantic labels, an alternative interpretation might be that changes to PPA activity related to improvements in aerial scene recognition reflect associative learning, proposed to be supported by parahippocampal cortex (PHC) (Aminoff et al. 2013). That is, participants may have learned to associate aerial images with their semantic labels, relying on preexisting connections with terrestrial representations, rather than accommodating novel scene perspectives into existing scene representations. This question resembles ongoing debates concerning the function of PHC, particularly PPA. Some studies suggest PHC supports contextual processing and episodic memory (Bar et al. 2008; Aminoff and Tarr 2015), yet others have questioned the reliability of these effects (Epstein and Ward 2010), instead, stressing the uniqueness of posterior PHC (i.e., PPA) in supporting spatial processing (Epstein and Baker 2019). Thus, we acknowledge associative learning as a possible account, yet stress that the current study was not designed to differentiate between the two accounts, as its main goal was simply to address a more primary question: to what extent is scene‐selective PPA sensitive to aerial scene information and how such sensitivity changes with experience.

Third, one may ask whether our results are the artifacts of enhanced attention to aerial scenes rather than increasing experience. Assuming people pay more attention to less encountered stimuli, it could perhaps be argued that paying more attention to aerial scenes in the control group led to the smaller reductions in response magnitude compared to the experimental group in scene‐selective cortex. However, we suggest that not to be the case. It is unlikely that the controls paid more attention to the presented scenes over the course of the study. We have used an orthogonal fixation‐cross task, which does not require controls to pay any attention to the scenes (Hansen et al. 2018; Harel et al. 2022). Moreover, behavioral performance in the controls worsened over time, indicating they were not able to recruit their attentional resources successfully (Figure S1). Our neuroimaging results also provide evidence against an attention account. Control regions in OTC (EVC and LOC) showed no distinct effects of training on the extraction of scene information over time, and their responses were uncorrelated with learning in the experimentals. The fact that training effects were relegated to scene‐selective cortex precludes the possibility that changes to scene‐selective cortex reflect a general attention effect. Such attentional effects tend to uniformly modulate neural activity across multiple areas in OTC and are not specific (Harel et al. 2010, 2013a, 2014).

The current study also reveals that naturalness and viewpoint are processed differently across OTC, irrespective of training. Scene‐selective PPA and OPA were sensitive to both scene properties, evincing stronger responses to terrestrial—compared to aerial scenes, and man‐made—compared to natural scenes. LOC activity was indifferent to viewpoint, responding only to naturalness, in line with previous research (Park et al. 2011). And although EVC was sensitive to naturalness and viewpoint, it showed a viewpoint effect in an opposite direction to scene‐selective cortex: aerial scenes evoked stronger activation than terrestrial scenes, and this “opposite” viewpoint effect was observed only for the natural, not man‐made, scenes. The finding that scene properties are processed differentially in scene‐selective cortex compared to EVC demonstrates that information represented in scene‐selective cortex is not inherited from EVC and is uniquely processed (Kohler et al. 2013; Kravitz et al. 2011).

Despite the strong similarities between them, OPA and PPA did differ in the way viewpoint information was modulated by naturalness. Whereas in OPA the viewpoint effect was stronger in the natural scenes compared to the man‐made scenes, in PPA the viewpoint was relatively stronger for the man‐made scenes than for the natural scenes. Although speculative at this point, this difference might be related to differential retinotopic biases within the scene‐selective network. OPA is reported to show a lower visual field bias, whereas PPA displays an upper visual field bias (Silson et al. 2015). These differential visual field representations are suggested to support functional differences, with OPA responsible for processing navigation‐relevant local scene information, and PPA for processing global scene structure for scene categorization (Dilks et al. 2021). Thus, integration of viewpoint information might be related to these retinotopic biases, with varying scene image statistics underlying the man‐made‐natural distinction (e.g., texture, rectilinearity, orientation, and spatial coherence) utilized differentially by scene‐selective regions (Groen et al. 2017, 2022). Further, in terms of division of labor between scene‐selective regions, the effects of scene properties and categorization training were manifest most strongly in PPA. This is consistent with PPA's putative role in scene categorization (Walther et al. 2009; Persichetti and Dilks 2019). Future studies using the current set of corresponding aerial and terrestrial scenes but utilizing different training regimens and task goals will establish how different types of experience impact different regions in the scene‐selective network.

In sum, our research highlights the utility of studying scene categorization training as a means of assessing the impact of experience on the scene‐selective network. The study provides novel evidence for the flexibility of the scene‐selective cortex (particularly PPA). Scene‐selective regions support learning of novel spatial geometries, accommodating for novel visual input through the activation of higher level semantic representations, which in turn modulate upstream perceptual processes.

## Author Contributions

**Joseph Borders:** conceptualization, investigation, data curation, formal analysis, visualization, writing – original draft, writing – review and editing. **Birken Noesen:** conceptualization, software, investigation, formal analysis, writing – original draft. **Bethany Dennis:** investigation, formal analysis, visualization, writing – original draft. **Assaf Harel:** conceptualization, investigation, formal analysis, visualization, writing – original draft, writing – review and editing.

## Ethics Statement

This study was performed in line with the principles of the Declaration of Helsinki. Approval was granted by the Wright State University Institutional Review Board. Informed consent was obtained from all individual participants included in the study. All identifying information has been deidentified.

## Conflicts of Interest

The authors declare no conflicts of interest.

## Supporting information


**Table S1:** Mean voxel count and standard error by ROI across groups.
**Table S2:** Full results of the ANOVA for response magnitude.

**Figure S1:** Learning trajectories across training sessions as a function of scene naturalness (manmade and natural) and viewpoint (aerial and terrestrial) for the control group.

**Figure S2:** Learning trajectories (A) reaction time; (B) accuracy across training sessions as a function of naturalness and viewpoint.

## Data Availability

The data that support the findings of this study are available from the corresponding author upon reasonable request.

## Endnotes

^1^
For recent works on the development of the scene‐selective network, see Dilks et al. (2021).

^2^
An earlier version of this study can be found in Borders et al. (2020). Note, however, the previous work is not focused on the question of scene plasticity and its uniqueness to specific regions in OTC.
