# Supplementary material for: Training in the Categorization of Aerial and Terrestrial Scenes Differentially Impacts Scene‐Selective and Nonscene‐Selective Regions in Occipitotemporal Cortex
Source: Eur J Neurosci. 2026 Jul 2;64(1):e70599. doi: 10.1111/ejn.70599 (PMC13326516; doi:10.1111/ejn.70599)
Supplement: Supplementary file 1 — Table S1: Mean voxel count and standard error by ROI across groups. Table S2: Full results of the ANOVA for response magnitude. [file EJN-64-0-s003.pdf]

Text S1

Materials & Methods

Regions of Interest

S1 Table reports the mean ROI cluster size (voxel count ± SEM) for each ROI in both the experimental and control groups. ROI sizes were extracted and summarized separately for each ROI and group to provide transparency regarding potential differences in cluster extent. Although ROI size was not explicitly equated across groups and activation values were not normalized by ROI size, statistical comparisons indicated no significant differences in ROI size across ROIs or between groups (all *p*s > .15).

Table S1. Mean Voxel Count and Standard Error by ROI Across Groups

	EVC	LOC	OPA	PPA
Experimental	3817 (592)	4718 (575)	4367 (492)	3650 (335)
Control	4863 (428)	3890 (407)	4380 (550)	3830 (408)

S2 Table reports the full list of all statistical analysis on the ROIs.

Table S2. Full results of the ANOVA for response magnitude.

Factor	df	Mean Squared Error (Greenhouse-Geisser)	F	Significance
Session	3,111	2.189	11.577	< .001
Session x Group	3,111	.012	.064	.979

ROI	3,111	34.624	73.346	< .001
ROI x Group	3,111	.310	1.015	.389
Naturalness	1,37	8.265	700.928	< .001
Naturalness x Group	3,111	.013	1.099	.301
Viewpoint	1,37	1.181	95.958	< .001
Viewpoint x Group	1.37	.128	10.424	.003
Session x ROI	9,333	.221	2.905	.003
Session x ROI x Group	9,333	.16	.400	.935
Session x Naturalness	3,111	.028	4.928	.003
Session x Naturalness x Group	3,111	.014	2.42	.07
ROI x Naturalness	3,111	.284	51.184	< .001
ROI x Naturalness x Group	3,111	.008	1.361	.258
Session x ROI x Naturalness	9, 333	.007	2.771	.014
Session x ROI x Naturalness x Group	9,333	.002	1.507	.144
Session x Viewpoint	3,111	.003	.550	.649
Session x Viewpoint x Group	3,111	.011	1.799	.152
ROI x Viewpoint	3,111	.993	92.18	< .001
ROI x Viewpoint x Group	3,111	.027	3.176	.027
Session x ROI x Viewpoint	9,333	.011	1.447	.231
Session x ROI x	9,333	.004	1.587	.118

Viewpoint x Group				
Naturalness x Viewpoint	1,37	.074	14.507	< .001
Naturalness x Viewpoint x Group	1,37	.002	.307	.583
Session x Naturalness x Viewpoint	3,111	.0001	.089	.966
Session x Naturalness x Viewpoint x Group	3,111	.028	6.635	< .001
ROI x Naturalness x Viewpoint	3,111	.057	28.465	< .001
ROI x Naturalness x Viewpoint x Group	3,111	.002	.826	.482
Session x ROI x Naturalness x Viewpoint	9,333	.002	.756	.586
Session x ROI x Naturalness x Viewpoint x Group	9,333	.001	.421	.923

Control Group Task

As described in the main text, to examine whether the training effects observed in the scene categorization training paradigm did not simply reflect the impact of repeated exposure to the scene stimuli, we included a control group of participants who viewed the same scene stimuli the same number of times, across the same number of sessions (including the sixth session to assess learning transfer) as the experimental group. Control participants performed an orthogonal, passive fixation cross task overlaid on the scenes that did not require active involvement with the scene content for the first five training sessions. Specifically, the participants performed an

orthogonal task in which in each trial, a centrally-presented fixation cross changed either in length or width (taller or wider, respectively) relative to an equal-hand fixation cross presented before the onset of the image, that is, during the inter-trial interval. Participants were asked to report by button press whether the horizontal or vertical bar of the central fixation cross lengthened. Note that the change was pseudo- randomized across all trials and hence was independent from the actual content of the underlying image, requiring the participants to pay very little, if any, attention to the background images. This task was previously employed in prior studies using scene stimuli (Nador et al., 2022; Hansen et al., 2018; Harel et al., 2016; Kravitz et al., 2011). Critically, the only factor differentiating the experimental from the control group was the task they performed: whereas the former actively categorized the scenes, the latter performed an orthogonal, passive fixation cross task that did not require any active involvement with the scene content.

Results

Control Group Behavioral Performance (IES)

Assessing learning during the first five sessions, we found a significant main effect of Session (F(4,80) = 3.99, $p < .05$, $\eta_p^2 = .15$). However, the pattern of performance over time of the control group was quite distinct relative to the experimental group (Figure S1, cf. Figure 2). While control participants' performance improved at the second session, it actually declined with each subsequent session, fitting a quadratic effect ($F(1,20) = 17.48$, $p < .001$, $\eta_p^2 = .47$). In fact, performance at the fifth session ($M = 643$, $SEM = 48.83$) did not significantly differ from baseline performance at first session 1 ($M = 562$, $SEM = 17.07$; $t(20) = -1.83$, $p = .08$). It is also

important to note there was a lot more variation in responses in the later sessions, with SEMs linearly increasing over the course of the training, potentially reflecting fatigue and/or carelessness when performing the fixation cross task. Notably, this trend is not observed with the experimental group.

-- Insert Figure S1 --

As additional evidence for the lack of any substantial learning effects, we did not observe any significant interaction effects of Session with any of the scene properties (either two-way or three-way, all *p*'s > 0.50), indicating that the extraction of scene information did not vary over time. Control participants completed the fixation cross task faster in the natural scene trials (*M* = 572, *SEM* = 27) compared to the manmade scene trials (*M* = 590, *SEM* = 28) (significant main effect of Naturalness: $F(1,20) = 150.68$, $p < .001$, $\eta_p^2 = .88$). Further, this Naturalness effect manifested differently across Viewpoint (significant Viewpoint-by-Naturalness interaction, $F(1,20) = 21.00$, $p < .001$, $\eta_p^2 = .51$). A post-hoc t-test revealed that participants performed the task significantly faster during the manmade aerial condition compared to the manmade terrestrial condition ($t(20) = -3.39$, $p < .003$), which is, in fact, in the *opposite* direction of the pattern observed in the experimental group. No significant difference between aerial and terrestrial scenes was observed for the natural scenes ($t(20) = -.75$, $p < 0.46$). We interpret these effects as likely to reflect incidental difficulties in discriminating the red fixation cross from its background, primarily in the manmade terrestrial scenes.

Finally, comparing performance between the first (*M* = 562 ms, *SEM* = 17) and sixth session (*M* = 632 ms, *SEM* = 49) also failed to yield significant differences ($t(20) = -1.45$, $p < .2$), ruling out the possibility that participants showed any learning transfer effects. Taken together, these findings support the notion that the training effects observed in the experimental group are specific and reflect the nature of the categorization task, rather than simply the effect of repeated exposure to the scene stimuli.

Experimental Group Behavioral Performance (RT and Accuracy)

Within Set Learning

Reaction Time. Reaction times showed evidence of learning across the five training sessions ($F(4,68) = 5.470$, $p < .001$, $\eta_p^2 = .243$). Categorization performance improved progressively, as indicated by a significant linear trend, $F(1,17) = 8.311$, $p < .05$, $\eta_p^2 = .328$). The most pronounced learning improvement occurred between the first training session (*M* = 950.332 ms, *SEM* = 56.757) and the second (*M* = 887.195 ms, *SEM* = 52.601) training sessions (Figure S2).

-- Insert Figure S2 –

Categorization performance varied as a function of scene properties. Participants categorized terrestrial scenes (*M* = 839.433 ms, *SEM* = 46.988) significantly faster than aerial scenes (*M* = 911.931 ms, *SEM* = 52.307), reflected in a main effect of Viewpoint, $F(1,17) = 33.064$, $p < .001$, $\eta_p^2 = .660$. Similarly, natural scenes (*M* = 844.766 ms, *SEM* = 49.759) were

categorized more quickly than manmade scenes (*M* = 906.598 ms, *SEM* = 49.335), as indicated by a main effect of Naturalness, $F(1,17) = 42.097$, $p < .001$, $\eta_p^2 = .712$). These two scene properties also interacted, $F(1,17) = 41.164$, $p < .001$, $\eta_p^2 = .708$, such that the advantage for terrestrial views was more pronounced for manmade scenes (Manmade Aerial: *M* = 960.288 ms, *SEM* = 54.233; Manmade Terrestrial: M = 863.573 ms, SEM = 50.992) ($t(17) = 7.50$, $p < .001$) than for the natural scenes (Natural Aerial: *M* = 852.908 ms, *SEM* = 45.308; Natural Terrestrial: *M* = 825.959 ms, *SEM* = 48.133) ($t(17) = 3.37$, $p < .005$) (Figure S2).

Accuracy. Categorization accuracy improved significantly across the five training sessions (main effect of Session: $F(4,68) = 26.123$, $p < .001$, $\eta_p^2 = .606$). This improvement followed a significant linear trend, $F(1,17) = 51.514$, $p < .001$, $\eta_p^2 = .752$, with the largest gain occurring between the first (*M* = .817, *SEM* = .013) and second (*M* = .871, *SEM* = .012) training sessions (Figure S2).

Performance also varied based on scene properties. Terrestrial scenes (*M* = .906, *SEM* = .01) were categorized more accurately than aerial scenes (*M* = .822, *SEM* = .011) (main effect of Viewpoint: $F(1,17) = 250.122$, $p < .001$, $\eta_p^2 = .936$). In addition, a significant interaction between Viewpoint and Naturalness emerged, $F(1,17) = 13.397$, $p < .005$, $\eta_p^2 = .441$). This interaction revealed that the accuracy gap was larger for natural scenes (Natural Aerial: *M* = .831, *SEM* = .009; Natural Terrestrial: *M* = .904, *SEM* = .01; $t(17) = 15.308$, $p < .001$) compared to the manmade scenes (Manmade Aerial: *M* = .813, *SEM* = .013; Manmade Terrestrial: *M* = .908, *SEM* = .011; $t(17) = 12.235$ $p < .001$).

Finally, the influence of the scene properties on categorization accuracy changed over the training sessions (Viewpoint by Naturalness by Session interaction: $F(4,68) = 3.095$, $p < .05$, η_p^2

= .154). Specifically, the extent to which participants grouped scenes from different viewpoints (aerial vs. terrestrial) evolved differently for manmade and natural scenes over training. For manmade scenes, categorization accuracy showed a significant effect of Session, $F(4,68) = 5.903$, $p < .001$, $\eta_p^2 = .258$). For natural scenes, this effect was also significant, though smaller, $F(4,68) = 2.834$, $p < .05$, $\eta_p^2 = .143$). Thus, with increasing training, the performance gap between aerial and terrestrial viewpoints of the same places was minimized (Figure S2).

Learning Transfer

Reaction Time. Next, we assessed the extent to which training effects transferred to novel scenes by comparing performance in Session 1, when participants categorized unfamiliar scenes with no prior training, to performance in Session 6, which also involved novel scenes but followed five sessions of categorization training. Reaction times for novel scenes were significantly faster after training than at the onset, $F(1,17) = 17.334$, $p < .001$, $\eta_p^2 = .505$).

Accuracy. Accuracy was significantly higher in Session 6 compared to Session 1, $F(1,17) = 31.355$, $p < .001$, $\eta_p^2 = .648$, indicating that the training regimen led to substantial improvements in the categorization of new, untrained scenes. Importantly, this improvement in generalization was modulated by the properties of the scenes, as indicated by a significant three-way interaction between Session (1 vs. 6), Viewpoint, and Naturalness, $F(1,17) = 7.22$, $p < .05$, $\eta_p^2 = .298$. This interaction suggests that the extent of learning transfer varied depending on whether participants were categorizing aerial or terrestrial views, and whether those scenes were manmade or natural.

List of Tables

Table S1. Mean Voxel Count and Standard Error by ROI Across Groups

Table S2. Full results of the ANOVA for response magnitude.

List of Figures

Figure S1. Learning trajectories across training sessions as a function of scene naturalness (manmade, natural) and viewpoint (aerial, terrestrial) for the control group

Figure S2. Learning trajectories (A. Reaction Time, B. Accuracy) across training sessions as a function of naturalness and viewpoint.
