# Supplementary figures and images for: Training in the Categorization of Aerial and Terrestrial Scenes Differentially Impacts Scene‐Selective and Nonscene‐Selective Regions in Occipitotemporal Cortex
Source: Eur J Neurosci. 2026 Jul 2;64(1):e70599. doi: 10.1111/ejn.70599 (PMC13326516; doi:10.1111/ejn.70599)

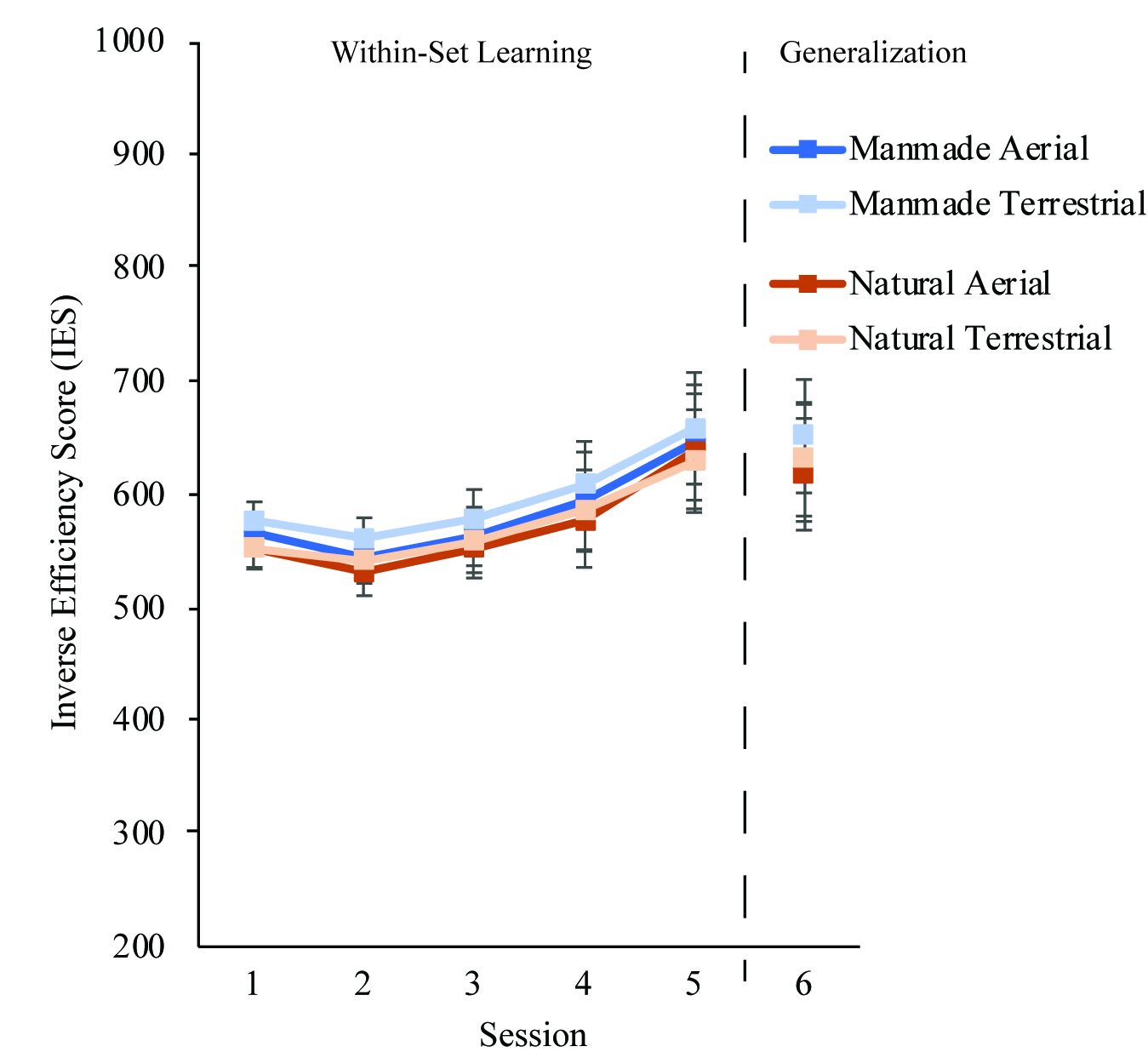

Supplement: Supplementary file 2 — Figure S1: Learning trajectories across training sessions as a function of scene naturalness (manmade and natural) and viewpoint (aerial and terrestrial) for the control group. [file EJN-64-0-s002.tif]

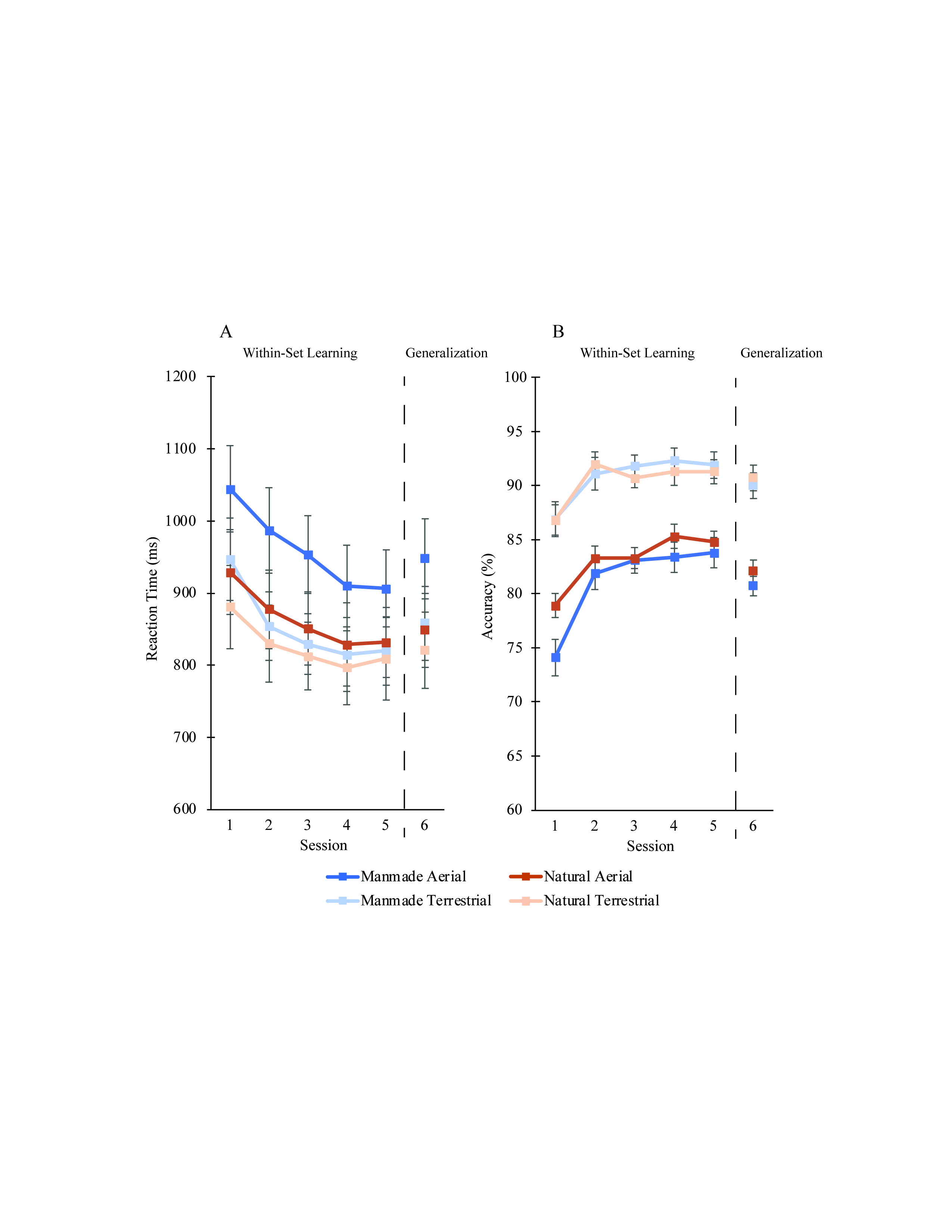

Supplement: Supplementary file 3 — Figure S2: Learning trajectories (A) reaction time; (B) accuracy across training sessions as a function of naturalness and viewpoint. [file EJN-64-0-s001.tif]
